# Quantitative Systems Pharmacology Models of Anti‐Amyloid Treatments for Alzheimer's Disease: A Systematic Review

**DOI:** 10.1002/psp4.70223

**Published:** 2026-03-05

**Authors:** Lara Herriott, Mark Coles, Nicolas Fournier, Eamonn Gaffney, Jonathan Wagg

**Affiliations:** ^1^ Mathematical Institute University of Oxford Oxford UK; ^2^ Kennedy Institute of Rheumatology University of Oxford Oxford UK; ^3^ AC Immune SA Lausanne Switzerland

**Keywords:** Alzheimer's disease, amyloid‐beta, monoclonal antibody, quantitative systems pharmacology, systematic review, virtual clinical trial

## Abstract

Quantitative systems pharmacology (QSP) models have emerged as useful tools for evaluating the efficacy of Alzheimer's disease (AD) therapies. Bringing together a clinical focus with the mechanistic detail of systems biology, QSP models are well suited to the complexity of AD and have been used to predict treatment outcomes and support regulatory submissions. Therapies targeting the amyloid pathway are prominent in the AD clinical trial landscape, with anti‐amyloid monoclonal antibodies representing the first approved disease‐modifying therapies. To inform and facilitate future QSP model development, a systematic review of published QSP models focused on amyloid‐targeting therapies for AD was completed. The PubMed and Web of Science databases were searched on February 1, 2025, identifying 540 candidate publications. Predefined exclusion and inclusion criteria were applied to identify seven published AD QSP models used to simulate treatment effects for one or more anti‐amyloid therapies. The structure, development, and predictions of the models were summarized. Shared and contrasting model features were identified across included models. A set of model quality features was scored against a checklist of 15 criteria adapted from “best practice” guidelines for QSP. Model quality scores were generally low, ranging from 40% to 53%. Key quality issues related to model validation and reproducibility were identified; in particular, none of the seven papers provided executable model code. This systematic review provides useful context to support ongoing efforts to develop and refine QSP models such that they may better inform therapeutic strategies for the treatment of AD.

## Study Highlights

- What is the current knowledge on the topic?
  ○ Quantitative systems pharmacology (QSP) models are valuable tools for evaluating Alzheimer's disease (AD) therapies, particularly anti‐amyloid treatments, and have been used to predict treatment outcomes and support regulatory submissions.
- What question did this study address?
  ○ This study systematically reviewed published QSP models focused on amyloid‐targeting AD therapies to ask to what extent they adhere to best practice guidelines, and what can be learned from their structural and predictive characteristics.
- What does this study add to our knowledge?
  ○ The review identified seven relevant QSP models and highlighted the wide range of model applications as well as key opportunities for improving model development, especially regarding reproducibility and extending model scope.
- How might this change drug discovery, development, and/or therapeutics?
  ○ By identifying strengths and weaknesses in current QSP models, the results of this systematic review can help inform future model development to enhance reliability, thereby improving the use of QSP in guiding AD therapeutic strategies.

## 1 Introduction

The recent approval of the first disease modifying therapies (DMTs) for Alzheimer's disease (AD)—the anti‐amyloid monoclonal antibodies (mAbs) aducanumab, lecanemab, and donanemab—marked a turning point in the field following decades of drug development without any new drug approvals [1]. However, the modest cognitive benefits observed with these treatments, combined with safety concerns surrounding potentially fatal brain swelling (ARIA‐E) and bleeding (ARIA‐H), have led to some restrictive approvals.

Although the US Food and Drug Administration (FDA) approved lecanemab, the European Medicines Agency (EMA) initially did not [2], citing an unacceptable risk–benefit balance. This decision was later updated, with lecanemab now approved only for APOEϵ4‐negative or heterozygous patients who are at lower risk of ARIA, bringing it in line with the approval of donanemab in the same patient subgroup.

The limited benefits and safety concerns of recently approved mAbs have prompted investigations into optimizing treatment approaches by altering dosing regimens, administration methods, and target populations. Opportunities for earlier intervention (e.g., for primary and secondary prevention) as well as combination therapies targeting additional AD pathways are also being actively explored.

Exploring such modifications to existing treatments offers significant scope to apply mathematical modeling. The paradigm of model informed drug development (MIDD) is grounded in the idea that by combining information from a range of sources, from in vitro to clinical studies, we may better understand the disease and its treatment, creating opportunities to streamline drug development and reduce drug attrition rates [3].

Here we present a systematic review of quantitative systems pharmacology (QSP) models, an emerging class of mathematical models that have been used to simulate anti‐amyloid AD therapies. QSP models bring together the clinical focus of pharmacokinetic/pharmacodynamic (PKPD) modeling, the method of choice for evaluating and understanding treatment efficacy and safety across populations, with the mechanistic detail of systems modeling. In doing so, QSP aims to generate more robust predictions, particularly in clinical contexts different from those in which the underlying model development data were generated, such as new patient populations, dosing regimens, or disease stages. The emerging field of QSP was first formalized in a 2011 NIH white paper [4] that stated: “QSP aims to develop formal mathematical and computational models that incorporate data at several temporal and spatial scales; these models will focus on interactions among multiple elements (biomolecules, cells, tissues, etc.) as a means to understand and predict therapeutic and toxic effects of drugs”.

The systems viewpoint that is intrinsic to the design of QSP models is particularly relevant to multifactorial diseases like AD [5], where genetic, environmental, and age‐related factors all contribute to complex pathophysiology across multiple scales. In addition, AD is poorly represented by animal models, which generally focus on individual aspects of disease pathology and lack other important features of human AD pathophysiology [6]. These features further motivate the development of QSP models to support clinical studies in AD.

In general, QSP models predict treatment outcomes by representing both the drug's mechanism of action and the underlying biological pathways that connect the drug's target to clinically relevant outcomes. In the context of AD, these clinically relevant outcomes include measures of cognition and function, as well as evidence of amyloid lowering now accepted as a surrogate endpoint for anti‐amyloid mAbs, meaning a reduction in amyloid is considered “reasonably likely to predict” clinical benefit [7]. A patient's amyloid burden can be assessed in vivo using positron emission tomography (PET) imaging and is quantified through metrics such as the standard uptake value ratio (SUVr). SUVr compares the amyloid PET signal in cortical regions known to accumulate amyloid with generally amyloid‐free reference regions such as the cerebellum. Modeling these image‐based metrics is therefore an increasingly relevant goal of QSP.

**FIGURE 1:**
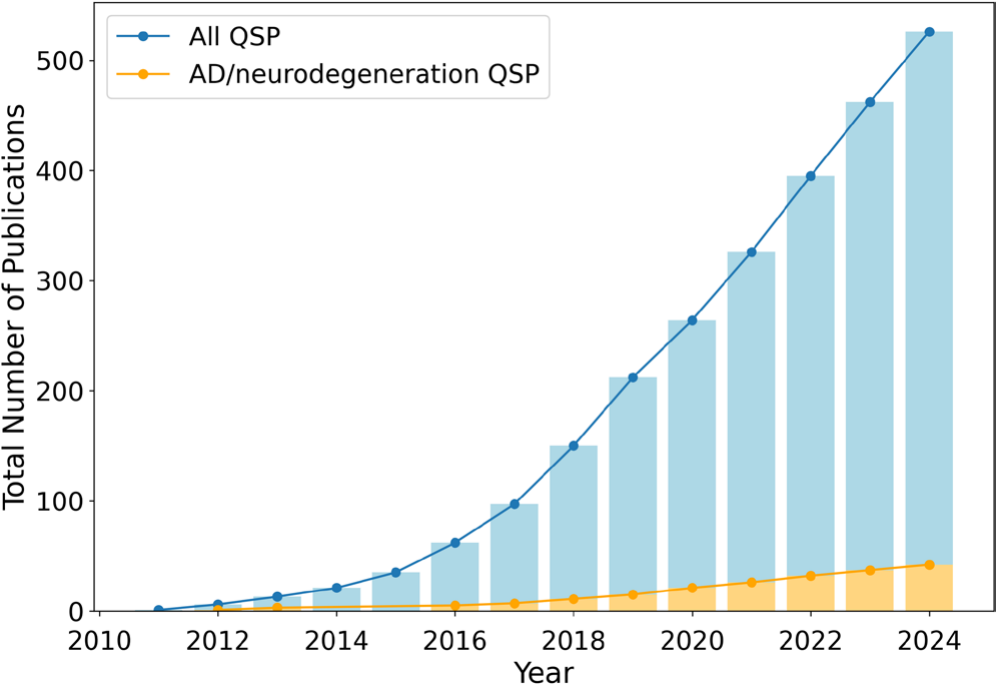
The number of published QSP models (blue), including for neurodegenerative disorders specifically (orange), has increased since 2011. Publications were identified by searching Scopus on October 15th, 2024. Search terms are provided in the Supporting Information Section S1.1.

Since 2011, the total number of published QSP models has steadily increased, as has the number of published QSP models of neurodegenerative disorders specifically, including AD (Figure 1). QSP models were first used in a regulatory setting in 2013 [8], and the inclusion of QSP models in FDA submissions has increased since then. Across all therapeutic areas, 157 FDA submissions utilized QSP modeling between 2013 and 2020, of which almost 60 were submitted in 2020 alone [9].

Despite this growth, the regulatory landscape around QSP modeling is less well developed compared to classical PKPD modeling, likely owing to a combination of the former's more recent uptake and greater complexity. Opportunities for regulatory engagement with QSP models have arisen through the FDA's “Model‐Informed Drug Development Paired Meeting Program” [10]. However, while the expectations for classical PKPD models were formalized in 2003 [11], no such formal guidance for QSP has yet been published. This relative immaturity of QSP as a field also gives rise to much variability in model development and terminology [12, 13, 14]. For example, the UK QSP Network stated that “the lack of standardisation in the way that quantitative and systems pharmacology (QSP) models are developed, tested, and documented hinders their reproducibility, reusability, and expansion or reduction to alternative contexts.” [12].

Most published models are also developed de novo, rather than by extending existing models [12, 13]. To address these issues of standardization and reproducibility, guidelines for best practices or suggested methods for various stages of model development have been presented [12, 15]. Others have gone further, proposing the development of a “clinical pharmacology backbone”—a collection of collaboratively developed, validated, and standardized models for application to drug development [16].

The lack of standardization across QSP models in general, combined with the potential of such models to build on the recent success of anti‐amyloid mAbs, underlies our motivation to systematically review QSP models of anti‐amyloid AD treatments. We follow the Preferred Reporting Items for Systematic Reviews and Meta‐Analyses (PRISMA [17]) checklist as closely as possible, with modifications where necessary to allow the application of review techniques to mathematical models. By summarizing and comparing the models published to date, a systematic review will help to flag areas requiring further development, minimize unnecessary reproduction of existing models, and foster collaborative work going forward.

Mathematical models of AD have recently been reviewed by Moravveji et al. [18]. Their scoping review identified a number of trends related to model quality, with only two of the 17 included studies meeting the validation criteria used. We therefore aim to evaluate QSP model quality with reference to structural and practical identifiability, parameter estimation, sensitivity analysis, and model validation, allowing us to identify the strengths and limitations of existing models and thereby promote more robust and reliable model development and analysis.

We focus here on a narrower class of model than those reviewed by Moravveji et al. [18], restricting the scope to QSP models of anti‐amyloid treatments specifically. This approach allows us to more clearly highlight common features across model structures, the processes involved in model development, and the predicted effects of various anti‐amyloid therapeutics. In this way, we can clearly demonstrate the importance of standardization, reproducibility, and collaboration.

## 2 Methods

### 2.1 Search Strategy

PubMed and Web of Science were both searched with no date restrictions. The search terms developed concern the three main aspects defining the scope of the review: Alzheimer's disease, mathematical models, and treatments. The specific terms were designed to be broad and inclusive of commonly used synonyms. Regarding “Alzheimer's disease,” we also consider reference to either amyloid or tau in the title. For “mathematical model” we consider “quantitative systems pharmacology” specifically, as well as terms such as “in silico” and “simulation.” For treatment, synonyms such as intervention, agent, and drug were applied. Finally, the search was modified to exclude publications that refer exclusively to animals, applying search terms developed in van der Mieden et al. [19]. No language or publication type limits were applied. Searches were conducted on February 1, 2025. The full search terms for both databases are provided in Supporting Information Sections S1.2 and S1.3.

### 2.2 Study Selection

Duplicates were removed using Rayyan's built in duplicate detection software [20] in two stages. First, any papers with exactly matching titles were removed algorithmically. Any remaining potential duplicates detected by Rayyan were then filtered manually. Screening of publications by a single reviewer proceeded in three levels: title screening, abstract screening, and full text screening. The exclusion criteria were: machine learning models; animal, cell, or in vitro models; mathematical models developed to simulate animal, cell, or in vitro experiments; mathematical models at the scale of individual molecules (e.g., molecular docking, molecular dynamics) or single cells; epidemiological models; reviews; and conference proceedings.

The inclusion criteria were: systems pharmacology models of AD; and simulation of an anti‐amyloid treatment. We draw on the definition of QSP models proposed in the NIH White paper [4] to inform the “systems pharmacology models of AD” criterion, applying the following specific inclusion criteria to be able to clearly delineate study inclusion: (1) the model incorporates the function of molecules across different tissues (often represented as different compartments); (2) the model has a clinically relevant output, such as a biomarker readout; and (3) the model explicitly represents drug distribution and the drug's effect via its mechanism of action. “Anti‐amyloid treatments” may include biologics, small molecules, or active immunotherapies targeting components of the amyloid pathway.

### 2.3 Data Extraction

Data extraction was performed simultaneously with full text screening. For models that met the inclusion criteria, information on model structure and scope, model development, and the model‐based predictions were extracted. Data extraction was conducted using a standard form for all papers, and the supplementary materials were screened as well as the main manuscript. The full data extraction table, demonstrating the form used, can be found in Table S4.

### 2.4 Quality Assessment

The PRISMA protocol for systematic reviews calls for an assessment of study bias; however, such a concept is not directly applicable to mathematical models. Instead, we conduct a quality assessment of the included models, guided by the “best practices” for QSP models established by the UK QSP Network [12]. This “best practice” workflow outlines six stages for maximizing the use and re‐use of QSP models, covering: (1) Purpose and context of the model; (2) Model structure and modeling methodology; (3) Input data, knowledge, and assumptions going into the model; (4) Model verification; (5) Model validation; and (6) Model results, application, and impact. We derive a checklist of 15 model features applicable to the various stages of the “best practice” workflow, with the number of checked criteria being used as our model quality score (see also Table S5).

For “Purpose and context of the model” we require (i) a statement of rationale for the chosen modeling framework. For “Model structure and modelling methodology” we consider (ii) the inclusion of a model schematic, (iii) the statement of the mathematical equations, (iv) the variables, and (v) the initial/boundary conditions, together with details of the (vi) software and (vii) algorithms used to implement and solve the model. For “Input data, knowledge, and assumptions going into the model” we identify (viii) any discussion of assumptions and simplifications, (ix) the methods used for parameter fitting and (x) the resulting parameter values. For “Model verification,” which assesses the self‐consistency of the model and whether it is structurally “fit for purpose,” descriptions of (xi) identifiability, (xii) sensitivity, and (xiii) steady state analyses are considered. For “Model validation” we identify (xiv) whether validation is described, including a description of the data used. Finally, for “Model results, application, and impact” we check whether (xv) executable code is provided. These selected criteria are not an exhaustive checklist for model development, but represent a core set of processes which should be described or made available for a published model in line with established “best practices.”

### 2.5 Model Analysis

Considering the anti‐amyloid QSP models included in the review, we assess both quantitative and qualitative model features. Quantitatively, we compare the range of estimated values of shared parameters and initial conditions. Qualitatively, we assess similarities and differences in model structure and model development, alongside the outcomes predicted by the model. Where possible, we also seek to connect differences in model structure with differences in model‐based predictions. In addition, we consider what avenues for future development are suggested.

## 3 Results

### 3.1 Screening

**FIGURE 2:**
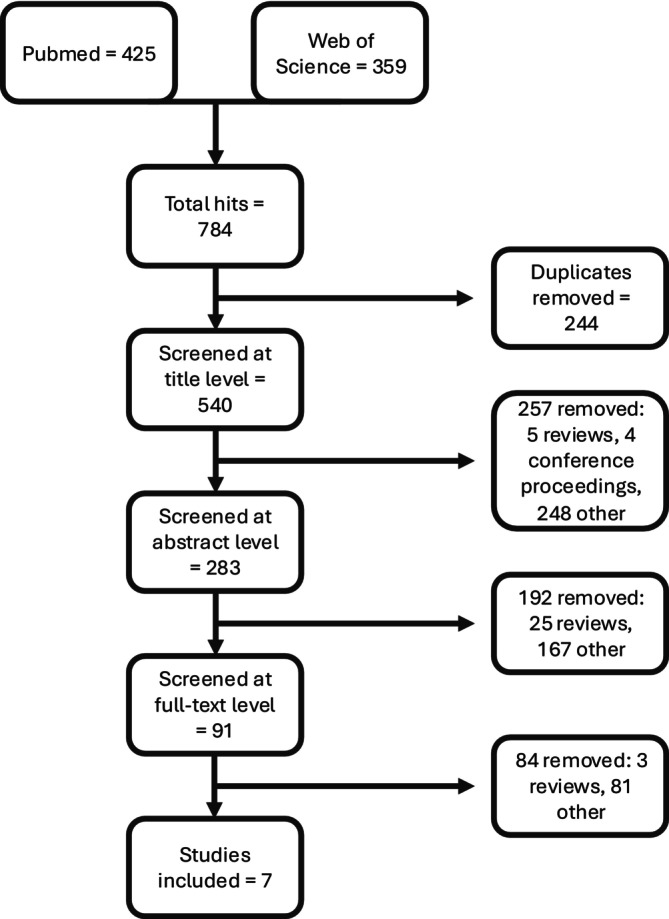
Literature screening flow chart highlighting the number of publications excluded at each stage of screening. Screening proceeded in three stages: title, abstract, and full‐text screening, with predefined inclusion and exclusion criteria applied at each stage. Overall, we identified seven anti‐amyloid QSP models for analysis in the systematic review, from an initial 540 unique publications.

The literature search identified 425 publications from PubMed and 359 from Web of Science, giving a total of 784 publications (Figure 2). After removing duplicates, 540 remained. During title screening, 257 publications were excluded, including 179 concerning molecular modeling and 46 referring to model organisms, leaving 283 papers taken forward to abstract screening. A further 192 papers were excluded at this stage, with the majority again concerning molecular modeling, leaving 91 for full text screening.

Full text screening identified seven anti‐amyloid QSP models meeting the inclusion criteria [21, 22, 23, 24, 25, 26, 27]. Full details on the screening results are provided in Supporting Information Section S2, and in Tables S1 and S2. Brief summaries of the seven anti‐amyloid QSP models that met the inclusion criteria are provided in Table 1 and in Supporting Information Section S3.

**TABLE 1:** Summary of the seven anti‐amyloid QSP models identified in the systematic review and their structure and application.

Model label	Publication	Description
1	Geerts et al. [20]	Compartmental ODE model to simulate amyloid aggregate clearance from the brain and ARIA‐E incidence as a result of anti‐amyloid mAb treatment
2	Ferl et al. [21]	Compartmental ODE model to directly compare the ability of two anti‐amyloid mAbs to neutralize amyloid oligomers in the brain
3	Markovic et al. [22]	Compartmental ODE model to simulate the effect of monomer‐binding mAbs on amyloid aggregation and plaque clearance from the brain
4	Lin et al. [23]	Compartmental ODE model to simulate the ability of aducanumab to clear amyloid plaque from the brain
5	Ramakrishnan et al. [24]	Compartmental ODE model to simulate amyloid aggregate clearance from the brain as a result of anti‐amyloid mAb treatment
6	Geerts et al. [25]	The same compartmental ODE as in model 1, additionally simulates AD in Down's Syndrome and an additional anti‐amyloid mAb
7	Madrasi et al. [26]	Compartmental ODE model to simulate the ability of anti‐amyloid mAbs to clear amyloid plaque from the brain and the effect of small molecule inhibitors on relevant biomarkers

*Note:* Full paper titles are provided alongside additional model descriptions in the Supporting Information.

Abbreviations: mAb, monoclonal antibody; ODE, ordinary differential equation.

Though excluded from the systematic review itself, publications that met two of the three inclusion criteria (being systems models with clinically relevant outputs; see Section 2.2 for further detail) can serve as useful reference models for future model development. Often, such models include a wider range of pathological AD pathways including tau, microglia and inflammatory factors as well as amyloid. We therefore provide a reference list of these 36 models in Table S3, categorized according to modeling methodology (e.g., network, QSP, classical PKPD) and treatments simulated. Overall, this list included seven QSP models for treatments targeting other aspects of disease pathology and eight classical PKPD models for various drugs.

This list also includes 15 models that did model anti‐amyloid interventions, but did so in ways that did not meet the strict inclusion criteria defined for this review. Namely, they did not model the treatments mechanistically, generally doing so by scaling parameter values instead. For example, the model of [28] simulates the anti‐amyloid mAb solanezumab, the gamma‐secretase inhibitor (GSI) semagacestat, and the beta‐secretase inhibitor verubecestat (BACEI) by directly suppressing Aβ42 levels by ~40%, ~20%, and ~70%, respectively.

### 3.2 Model Structure and Scope

All seven models included in this systematic review focused on the amyloid hypothesis of AD. We first considered their representations of amyloid processing and aggregation, non‐amyloid pathways, the different compartments used, and the populations represented. These features are summarized in Table 2, alongside model outputs and treatments simulated.

**TABLE 2:** Data extracted from the seven publications on model structure, scope and simulations.

Model label	1	2	3	4	5	6	7
*Compartments*							
Brain ISF	✓	✓	✓	✓	✓	✓	✓
CSF	LV, TFV, CM, SAS	✓	X	✓	✓	LV, TFV, CM, SAS	✓
Plasma	✓	✓	PK only	✓	✓	✓	✓
Lymph	✓	X	X	X	X	✓	X
Peripheral	✓	X	PK only	✓	X	✓	✓
*Population*							
Prodromal AD	✓	X	✓	✓	X	✓	✓
Early AD	✓	X	✓	X	X	✓	X
Mild AD	✓	✓	✓	✓	X	✓	✓
Moderate AD	✓	✓	✓	✓	X	✓	✓
MCI	✓	X	X	X	✓	✓	X
APOEε4	✓	X	X	X	✓	✓	X
Down's syndrome	X	X	X	X	X	✓	X
*Relevant amyloid biomarker*							
Amyloid PET (SUVr)	✓	X	✓	✓	✓	X	✓
Amyloid PET (CL)	X	X	X	X	X	✓	X
CSF Aβ concentration	✓	✓	X	X	X	✓	✓
CSF Aβ42 concentration	X	X	X	X	✓	X	X
Plasma Aβ concentration	X	✓	X	X	X	X	✓
Plasma Aβ42/40 ratio	X	X	X	X	X	✓	X
*Treatments*							
Aducanumab	✓	X	✓	✓	✓	✓	✓
Bapinezumab	✓	X	✓	X	X	X	✓
Crenezumab	✓	✓	✓	X	✓	X	✓
Gantenerumab	✓	X	✓	X	✓	✓	X
Lecanemab	✓	X	✓	X	X	✓	X
Solanezumab	✓	✓	✓	X	✓	X	✓
Donanemab	X	X	X	X	X	✓	X
BACEI	X	X	X	X	X	X	✓
GSI	X	X	X	X	X	X	✓

*Note:* Check marks for the CSF compartments indicate the presence of a single, general CSF compartment. See Table 1 for the publication associated with each label.

Abbreviations: BACEI, beta‐secretase inhibitor; CM, cisterna magna; CSF, cerebrospinal fluid; GSI, gamma‐secretase inhibitor; ISF, interstitial fluid; LV, lateral ventricles; MCI, mild cognitive impairment; PET, positron emission tomography; SAS, sub‐arachnoid space; SUVr, standard uptake value ratio; TFV, third fourth ventricle.

The seven anti‐amyloid models were all compartmental ordinary differential equation (ODE) models, with all models including at least one compartment representing the brain interstitial fluid (ISF) and one representing the blood/plasma. All models, except model 3, also contained at least one cerebrospinal fluid (CSF) compartment. Two of these seven publications use the same underlying QSP model (models 1 and 6) but perform simulations of different anti‐amyloid treatments in different contexts and patient populations (see Section 3.5 for further information).

These seven models span multiple AD populations, ranging from prodromal AD, through mild cognitive impairment (MCI) and “early AD,” to moderate AD. In general, the population simulated is drug dependent, corresponding to the clinical trial population used for QSP model calibration. Overall, three of the models are applied to APOEϵ4 carriers vs. non‐carriers (models 1, 5, and 6), and one (model 6) is also applied to the Down's syndrome (DS) population.

**FIGURE 3:**
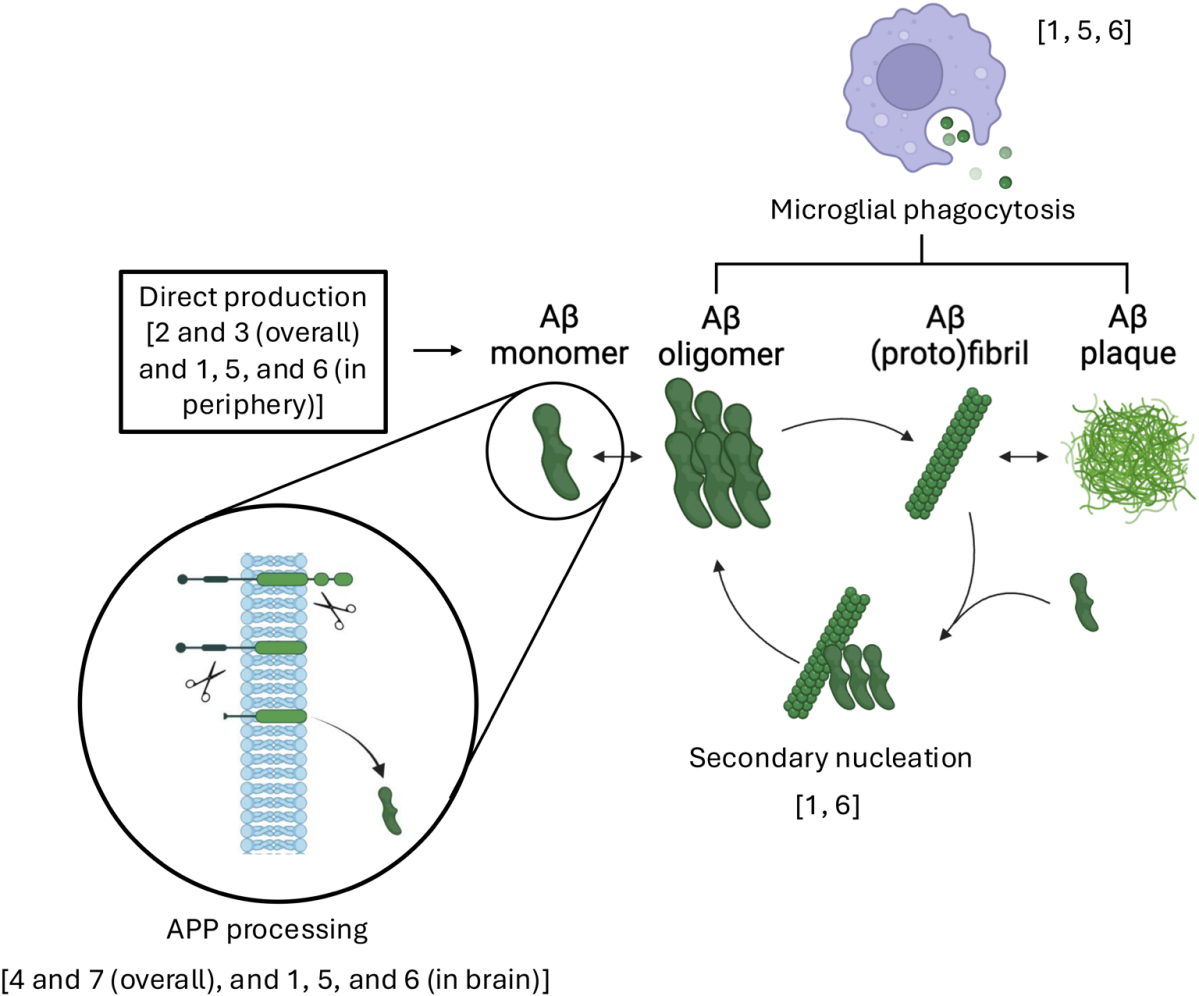
Overview of the amyloid pathway in AD, annotated with the models which include representations of direct Aβ production, APP processing, secondary nucleation, and microglial function. All models include representations of multiple amyloid species (including monomers, oligomers, (proto)fibrils, and plaque), with details of the species represented in each model presented in Figure 4. See Table 1 for the publication associated with each label. APP, amyloid pre‐cursor protein.

**FIGURE 4:**
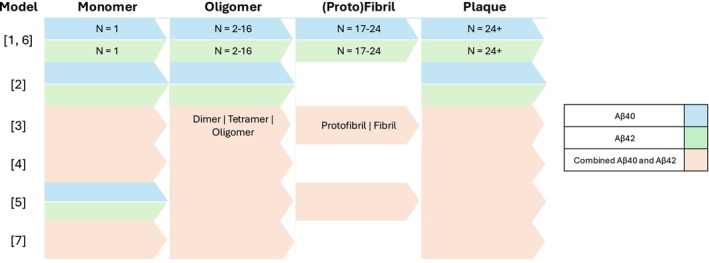
Overview of the amyloid aggregate species represented in each model arranged by amyloid aggregate size and colored by amyloid isoform represented: Aβ40 (blue), Aβ42 (green), or a combination of the two (orange). See Table 1 for the publication associated with each label.

Two of the seven models (models 4 and 7) represented brain Aβ production via APP synthesis (Figure 3), including beta‐ and gamma‐secretase cleavage, while two (models 2 and 3) modeled brain Aβ production directly. In models 1, 5, and 6 peripheral Aβ production is modeled directly while in the brain APP synthesis is modeled. The representation of Aβ aggregation ranged from explicit, with aggregates of sizes between 1 and 24 monomers represented separately (as in models 1 and 6) to coarse‐grained (Figure 4). A coarse‐grained representation comprising monomer, soluble oligomers, and insoluble plaque was the single most common structure, being used in three QSP models (models 2, 4, and 7). In these three models, the variables representing oligomer and plaque track the total mass of Aβ that exists within each size of aggregate. Model 5 takes a similar approach, but represents the higher‐order aggregates of fibrils and plaque in terms of how many oligomers (taken to be made up of 10 monomers) they contain. In contrast, models 1 and 6 instead track the number of aggregates of each size. Model 3 differs from all other models by using arbitrary concentrations for all aggregate species.

Out of all seven QSP models, three models (1, 2, and 6) represented Aβ40 and Aβ42 separately, while two considered a combined pool of Aβ40 and Aβ42 (models 4 and 7) and one modeled a single generic Aβ species (model 3) (Figure 4). Model 5 represented Aβ40 and Aβ42 monomers separately, but modeled a combined pool of oligomers and plaque. None of the models included aspects of tau pathology or neurodegeneration, although four publications mentioned the former as a possible avenue for future model extension (models 1, 4, 5, and 6).

Amyloid biomarkers, such as amyloid PET SUVr and plasma or CSF Aβ concentrations, were the clinically relevant endpoints connected to all models (Table 2), rather than direct clinical outcomes like the Clinical Dementia Rating Scale–Sum of Boxes (CDR‐SB) or the Alzheimer's Disease Assessment Scale–Cognitive Subscale (ADAS‐Cog). Six of the models focused on simulating a reduction in brain amyloid aggregate concentration as the primary output, but expressed this in terms of a reduction in amyloid PET signal to align with observed clinical biomarkers. In four models (models 3, 4, 5, and 7), the relationship between plaque burden and PET signal was assumed to be directly proportional, meaning a given percentage reduction in plaque (and fibril, in the case of model 5) translates to the same percentage reduction in PET signal. Alternatively, models 1 and 6 derived a model‐specific expression to relate the two changes. Only model 2 did not simulate a reduction in amyloid plaque, instead focusing on antibody‐driven oligomer clearance and relating model outputs to fluid biomarkers only.

The mechanism driving clearance of brain amyloid begins in all models with binding of antibody to the various amyloid aggregates according to the law of mass action. In models 2, 3, 4, and 7, the total mass of Aβ contained in each amyloid aggregate can be bound by antibody. In contrast, since model 5 considers higher‐order aggregates in terms of the number of oligomers they contain, only 1 in 10 Aβ molecules within these larger aggregates is available for antibody binding. In two models (2 and 3), the amyloid‐antibody complexes that form are then cleared directly (as described above, in model 2 only antibody‐oligomer complexes are cleared). In contrast, in models 4 and 7, the antibody‐amyloid complexes must first form additional complexes with Fc receptors (FcRs) before being cleared. This reflects the biological mechanism underlying mAb‐dependent plaque clearance, whereby activation of microglial FcRs stimulates phagocytosis.

Three of the models included an explicit representation of microglia and their role in amyloid clearance (models 1, 5, and 6; Figure 3). In models 1 and 6, microglia could exist in a high phagocytotic or low phagocytotic state, with Aβ‐antibody complexes promoting microglial proliferation and driving phenotypic switching from the low to the high phagocytotic state. In model 5, microglia were included following assessment of a previous model iteration in order to correctly predict amyloid clearance by various mAbs. No proliferation is modeled, but microglial activation is, with the presence of Aβ‐antibody complexes driving switching from a resting to an active phagocytotic state. In both models microglia clear both antibody‐bound and free amyloid, but at different rates.

### 3.3 Model Development

The processes of parameter estimation and model validation are critical stages in model development on which the reliability of model predictions depends. We therefore consider whether details on these stages are provided in the included publications, and evaluate the information where available (Table 3).

**TABLE 3:** Data extracted from the seven publications on key stages of model development: model fitting, simulation, and validation.

Model label	1	2	3	4	5	6	7
Fitting protocol	X	X	X	Simultaneous	Simultaneous	X	Iterative
Clinical trial data	✓	✓	✓	✓	✓	X	✓
Natural history	X	X	SUVr [29]	Steady states; CSF SILK	X	X	Steady states; CSF SILK
Software	X	X	R; Phoenix WinNonlin v8.3	Matlab 2017; KroneckerBio v0.5.1.1	MATLAB SimBiology v2017B	X	Matlab 2019a; KroneckerBio v0.5.2.3
Validation Data	X	X	X	Phase Ib aducanumab	Phase III aducanumab & gantenerumab; APOEε4 non‐carrier	Phase III donanemab & gantenerumab	X

*Note:* See Table 1 for the publication associated with each label.

Abbreviations: CSF, cerebrospinal fluid; LTE, long term extension; MAD, multiple ascending dose; PK, pharmacokinetic; SAD, single ascending dose; SILK, stable isotope labelling kinetics; SUVr, standard uptake value ratio.

Details on the data used for parameter estimation are provided for all models, although information on how parameters were estimated is present for only three of these models (models 4, 5, and 7). However, these descriptions of parameterisation are only indicative of the approach used, without stating the specific methods utilized. The data used for parameter estimation includes clinical trial data in all cases, and commonly a combination of clinical trial and other clinical or translational research data, such as that from stable isotope labelling kinetics (SILK) experiments conducted in AD patients.

Parameter values, including their units, are provided for all models, while the initial model variable values used to perform the presented simulations were described only for four models (2, 3, 4, and 7). Due to the high degree of structural similarity across a number of the models, we were able to directly compare a number of parameters (Table 4) and some values for model variable concentrations. While one model set initial concentrations to arbitrary values (model 3), the other models where this information was provided (models 2, 4, and 7) drew on relevant literature.

**TABLE 4:** Common parameter values compared across four models with sufficiently similar structures.

Parameter	Model 4	Model 7	Model 5	Model 2
Aβ plasma→csf	1.72E‐09	1.72E‐09		
Aβ csf → plasma	4.17E‐05	4.50E‐05		
Aβ brain→plasma	1.48E‐05	1.48E‐08	2.43E‐05	8.39E‐05
Aβ plasma→brain	1.48E‐04	1.48E‐07		
Aβ brain→csf	1.55E‐05	7.50E‐05	3.49E‐05	8.39E‐05
Aβ brain clearance	1.93E‐05	5.50E‐05	9.89E‐05	3.36E‐04
Aβ plasma clearance	9.63E‐05	1.90E‐04	6.42E‐04	9.49E‐03
Ab plasma clearance	1.46E‐06	1.50E‐07		
Ab plasma→csf	1.72E‐09	1.72E‐09		
Ab csf→plasma	4.17E‐05	8.00E‐06		
Ab plasma→brain	1.60E‐06	1.72E‐09		
Ab brain→plasma	3.20E‐03	8.10E‐06		
Ab brain→csf	1.55E‐05	3.20E‐06		
ADCP	3.60E‐03	3.70E‐05		

*Note:* See Table 1 for the publication associated with model. The first five parameters represent Aβ transport, the following two Aβ clearance, followed by an antibody (Ab) clearance parameter, five Ab transport parameters, and finally the rate of antibody‐dependent cellular phagocytosis (ADCP). All parameters are shown in s^−1^—where this was not the unit used in the model the parameters have been scaled appropriately. In both models 5 and 2, transport values are represented as fractions of clearance rates. These calculations have therefore been applied to obtain the values in the table. Full details are provided in Section S4.

Abbreviations: Ab, antibody; ADCP, antibody dependent cellular phagocytosis; CSF, cerebrospinal fluid.

First, comparing the almost identically structured models 4 and 7, we find that although the same publications are referenced for the steady state concentrations of amyloid species, the values used differ by up to 38‐fold. While the brain monomer concentrations are similar (0.98 and 0.75 nM in model 4 and 7, respectively), the chosen aggregate concentrations are markedly higher in model 4 than in model 7: brain oligomer steady state concentrations are 96 vs. 4.4 nM, while plaque steady state concentrations are 1500 vs. 39 nM, respectively.

Accordingly, differences in the transport parameters for amyloid transport to and from the brain are also observed for these two models (Table 4). Though the rates of transport from plasma to CSF and CSF to plasma are very similar, the plasma to brain and brain to plasma rates are three orders of magnitude lower in model 7 compared to those in model 4, though the resulting equilibrium constant is the same in both cases. Given the concentrations of amyloid aggregates in the brain were so much higher in model 4 compared to model 7, very different antibody:plaque ratios would be achieved if the same PK model was applied. Instead, we find a possibly compensatory difference in plasma to brain antibody transport between models 4 and 7. The two orders of magnitude difference in the rate of ADCP may also act to correct for the differences in concentration. We find that the brain to plasma rates in models 2 and 5, which are structurally similar to models 4 and 7, are most similar to those in model 4, all being of the order $10^{-5}\;s^{-1}$. Models 2 and 5 do not model any transport of amyloid from the plasma to the brain. In line with this assumption, the rates of plasma amyloid clearance in models 2 and 5 are higher than those in models 4 and 7.

The software used to implement and solve the models is stated in only four instances: MATLAB is used for three models (4, 5, and 7), of which two utilize KroneckerBio (models 4 and 7), while one uses R and Phoenix WinNonlin v8.3 (model 3).

Model validation is described explicitly in only three publications (4, 5, and 6). In all three cases, the validation included predicting percentage reductions in brain amyloid for either a drug or a dose that was not used during parameter fitting. The validation of model 6 effectively acts as validation for model 1, since it validated model predictions for donanemab, a drug that was not simulated in the first study as part of model calibration. In addition to validating model predictions of amyloid reduction, model 5 validated model‐predicted disease progression over 50 weeks by comparing simulations for APOEϵ4 non‐carriers with those for APOEϵ4 carriers, and model 4 validated a PK profile predicted by the model for a dose of aducanumab not used for model calibration.

### 3.4 Model Quality

Compiling the various model features that have been extracted, we formulated a model quality metric as described in the Methods (Table S5). All of the models had scores of 6/15 or higher, with two scoring 8/15, which was the highest.

Of particular note, none of the models satisfied the criteria for the algorithm used to solve the model, the details of parameter fitting, identifiability analysis, or the provision of code. In deciding whether details of parameter fitting were provided, we considered whether the information was sufficient to independently replicate the fitting procedures reported in each publication. Although, as stated above (Table 3), some reference to parameter estimation was provided, in all cases this was insufficient for reproduction of model calibration.

All publications provided an explanation of the rationale for the model, a schematic of model structure, and the model equations and parameter values.

Regarding sensitivity analysis, though this was presented for model 4, it was only a local analysis, not global. Comprehensive sensitivity analyses were not described in the other models; however, the sensitivity of the model to an individual parameter was reported in some cases. For example, the effect of varying the endogenous plaque turnover rate in model 7 was explained in the context of treatment with different drugs. As described above, only three of the models (4, 5, and 7) met the criteria regarding validation.

### 3.5 Model Predictions

In addition to reviewing model structure and development, we analyzed the simulations presented in the publications to understand how the models were applied to relevant research questions.

All models were used to simulate anti‐amyloid mAb treatment responses, and one publication (model 7) also simulated beta‐ and gamma‐secretase inhibitor treatment responses. Most models compared multiple mAbs, with only one publication presenting simulations for a single mAb (model 4). In total, seven different mAbs were simulated across all models: aducanumab, bapinezumab, crenezumab, donanemab, gantenerumab, lecanemab, and solanezumab.

These different mAbs are primarily distinguished by their binding affinities to different amyloid species. When multiple models simulated the same mAbs, generally consistent dissociation constant ($K_{D}$) values were used for the highest affinity mAb‐amyloid interactions (Table S6). However, $K_{D}$ values varied considerably for the lowest affinity interactions, where arbitrarily high $K_{D}$ values were chosen to represent negligible binding.

Consistent with brain amyloid clearance being the primary mechanism of action of many anti‐amyloid mAbs and an FDA‐accepted surrogate endpoint, all models simulated decreases in brain amyloid aggregate levels. A majority of models (6/7) connected percentage decreases in insoluble amyloid concentration (mainly of fibril and plaque) to observed decreases in amyloid PET signal as described in Section 3.2.

Fluid biomarkers were simulated less frequently, with 5/7 models simulating CSF amyloid concentration, 2/7 simulating plasma amyloid concentration, and only one simulating the plasma Aβ 42/40 ratio (1/7). Two models (models 1, 6) also simulated ARIA‐E incidence, thus capturing a clinically relevant safety metric.

The models were applied to a number of different simulation scenarios. Most commonly, biomarker changes were investigated over an 18‐month clinical trial duration. Evaluating the results of these simulations, we observed variation in the predicted amyloid plaque clearance trajectory. Aducanumab, for example, which was simulated by six of the models (models 1, 3, 4, 5, 6, and 7), was predicted to drive plaque clearance with variable dynamics: in model 1, clearance follows an apparently linear trajectory, while in model 5, exponential plaque clearance following a delay was predicted.

In addition to simulating the current, standard, 18‐month clinical trial duration, some publications simulated hypothetical longer‐term treatment durations of up to 10 years, either with the same dose as in the trial (e.g., model 4), or within the context of investigating maintenance treatment regimens (model 6). Model 6 also investigated how a hypothetical 3‐month treatment break, followed by treatment restart with dose titration back to the full dose, might reduce ARIA‐E incidence.

Other publications simulated hypothetical drugs with different properties to those developed to date: two of these (models 2 and 3) consider drugs with increased brain penetration and one (model 7) considers three hypothetical mAbs that exclusively bind either monomer, oligomer, or plaque. Another repeated application of the models was to simulate treatment responses in different AD populations. Differences between the APOEε + and—populations were investigated in a number of models (1, 5, and 6), with model 6 also presenting simulations for a DS population, modeled via a 1.5× increase in the rate of APP production.

Finally, two of the models were also used to simulate disease progression over periods ranging from 50 to 80 years (models 1 and 3), with only model 1 capturing the sigmoidal pattern of amyloid accumulation.

## 4 Discussion

### 4.1 Evaluation of Screening Criteria

In developing the inclusion criteria, we allowed for variation in how the term “quantitative systems pharmacology” is used across the literature [14], particularly that mathematical models could meet the requirements to be a QSP model without being labeled as such. We based our inclusion criteria on the definition for “QSP models” proposed in [4], requiring systems models of AD with clinically relevant outputs and anti‐amyloid treatments modeled mechanistically.

Mathematical models of AD, including those taking a systems approach, but which do not extend to therapeutic modeling, have been surveyed in a recent review by Maji and Khajanchi [30]. The specific criteria we applied to define QSP, with a focus on the modeling of anti‐amyloid interventions, led to the exclusion of a number of systems models of AD with clinically relevant readouts and which simulated anti‐amyloid treatments, but where the drug was not modeled mechanistically. In some cases, these models were described as QSP models. However, by simulating the treatment only by scaling a parameter, without connecting the drug's PK profile to its specific target, these models did not meet the criteria we established prior to conducting the literature search and screening. Although we acknowledge the exclusion of such models is a limitation of the approach taken, it was a necessary distinction to appropriately restrict the scope of the review, in particular to account for exclusion of models which simulate hypothetical treatments only.

Though models of this type did not reach the threshold for inclusion in the systematic review, they remain useful references for the development of QSP models, especially those that consider multiple pathological pathways. We have therefore separately documented these models in Table S3 for reference by modelers, alongside other mathematical models identified in the screening process which simulate treatments targeting other AD pathologies.

### 4.2 Model Quality, as Compared to Best Practice Guidelines, Revealed Opportunities to Improve Model Robustness

Variability in the definition of QSP in practice is to be expected given its more recent development as a field compared to other better‐established approaches such as classical PKPD modeling. Similarly, we observed much variation in the implementations of QSP models. In response to this variation, the UK Quantitative and Systems Pharmacology Network published “best practice” guidelines in an attempt to standardize QSP models [12], which were used to guide the quality assessment presented here.

One particularly striking finding from this review was the complete absence of identifiability analysis and the insufficient descriptions of parameter estimation. Model identifiability is a necessary condition for accurate parameter estimation: theoretically, a model is identifiable if precise parameter values can be inferred [31]. When developing a model, structural identifiability analysis should be used to determine whether, assuming perfect data, a unique set of parameter values exists given the model's structure, inputs, and observable outputs. In practice, however, especially in pharmacological modeling, the data is sparse and noisy, meaning we must also consider practical identifiability: given the actual data available, is it still possible to reliably infer this unique parameter set?

Our comparison of parameter values between models 4 and 7 highlights how practical identifiability issues can influence parameter estimates. We observed that the steady‐state concentrations of brain amyloid species, which were used as inputs for parameter estimation, differed substantially between the two models. These differences, in turn, led to differences in parameter values, as the models adjusted to compensate for variations in the input data.

The absence of structural identifiability can often be checked with relative ease as a routine step in model development. Simple methods such as the scaling invariance method (SIM) of Castro and de Boer can readily be applied [32]. It should be noted that this simple method only accounts for scaling symmetries which, although common, are not the only type of symmetry that can lead to non‐identifiability [33]. Despite this, the SIM remains a useful initial method for addressing structural identifiability, especially for non‐mathematicians [34], and it can be used to definitively demonstrate the absence of structural identifiability. Alternatively, methods such as profile likelihood could be used to check for structural and practical identifiability and to estimate confidence intervals on parameters.

Considering the insufficient descriptions of parameter estimation provided in the QSP models reviewed here, a particular concern is the potential for confusion over the dual use of the term “calibration” in QSP modeling. A recent review of QSP model validation distinguished between traditional methods of parameter estimation, such as least‐squares optimisation or Bayesian inference, and the process of “calibrating” a virtual population [35]. Calibrating a virtual population involves drawing samples from suggested biologically plausible ranges for each relevant parameter and identifying which parameter sets generate outputs in the range observed across a real population. The parameter sets which fulfill this criterion define the members of the virtual population. As a result, precise estimation of individual parameter values may be unnecessary, provided that by sampling across parameter uncertainties a virtual population that reproduces the observed data can be generated. The lack of detail on model calibration in the included studies is such that in many cases it cannot be determined whether virtual population calibration was used. Corresponding authors for all included publications were contacted seeking clarification on this matter, allowing confirmation that a typical approach to parameter estimation only, and not virtual population calibration, was conducted for models 2 and 5.

After model calibration, model validation allows the model's accuracy to be assessed within its intended context. Validation is especially important if the model will be extrapolated to settings beyond those used for model calibration. Given the challenges of sparse data and data availability in clinical settings, there may be limited scope to validate a model. Although the model can still be usefully applied despite these issues [9], it remains important to clearly set out at publication whether, and in what ways, a model has been validated.

A four‐tier framework has been proposed for discussing various approaches to validation [35]. Among other features, this framework distinguishes between model qualification (the ability to interpolate) and validation (the ability to extrapolate). Applying this framework to the publications included in this systematic review, we find one model at the “within target validation” level (model 4), with data for the same drug but at different doses being used for validation, and two at the “within pathway validation” level (models 5 and 6), where data for a different drug with a different amyloid binding profile were used. As described above, validation was not presented for the other models reviewed here.

### 4.3 Similarities and Differences in Model Structures, Applications and Predictions

We identified several components of the amyloid pathway and mechanisms of action of anti‐amyloid drugs that were consistently represented across the models reviewed, and thus serve as a useful framework for future QSP development. There was substantial overlap in the amyloid species represented in the seven models (specifically monomer, oligomer, and plaque), and also similarities in the inclusion of APP processing—where this was needed to accurately model beta‐ and gamma‐secretase inhibitors.

While the models approached consensus in their overall architecture, specific parameters varied substantially, reflecting the challenge of obtaining accurate experimental estimates from brain tissues.

In addition, none of the seven models reviewed included pathological changes upstream or downstream of amyloid. While this is appropriate for a group of models focused on the MCI disease stage and later, once amyloid pathology is already established, considering additional aspects of AD pathophysiology is important when seeking to apply QSP more widely. Specifically, factors upstream of amyloid would be relevant if investigating earlier interventions, and those downstream would allow simulation of therapeutics targeting tau or neuroinflammation.

The differing efficacies of anti‐amyloid mAbs were well captured by differing dose regimens and affinities to amyloid species. However, the observed variations in specific affinity parameters likely reflect differences in experimental assays and amyloid aggregate definitions. Similarly, the overall PK frameworks used were consistent, but with different parameterisations.

Although the overall PKPD relationships were consistent, with anti‐amyloid mAbs driving plaque reduction, our review identified differences in the representations of this antibody‐dependent clearance that related to the models' predictions.

In Section 3, both the different approaches to modeling plaque clearance and the different predictions in terms of the trajectory of plaque clearance were outlined. The former ranged from simply scaling up clearance of antibody‐amyloid complexes to modeling microglial activation and function, with the latter including predictions of apparently linear or exponential clearance trends. Cross referencing these features, we observe a somewhat counterintuitive trend where the more complex models, those including explicit representations of microglial function and allowing for clearance of both bound and unbound amyloid, are not able to generate the fast early dynamics of plaque clearance observed in clinical trials of lecanemab and donanemab. In comparison, the relatively simpler models, where clearance of antibody‐amyloid complexes only is allowed, do predict fast initial clearance.

Notably, early timepoint data was not used in the development of model 1. It is possible that more accurate predictions could be made if the model was re‐fitted to the results from the phase III CLARITY trial of lecanemab [36] which include 3‐ and 6‐month PET SUVr readouts, as well as 12‐ and 18‐month timepoints in line with the clinical trial results previously used for model development.

Overall, these observations suggest a gap between the models' clearance mechanisms and the biological reality. That the formation of antibody‐amyloid complexes activates microglia to promote aggregate clearance by ADCP is generally accepted as the main mechanism of action of the approved anti‐amyloid mAbs. It is also reasonable to suggest that once activated, the microglia act indiscriminately to clear plaque, phagocytosing both free and antibody‐bound amyloid aggregates. This is implied by the large degree of plaque clearance driven by donanemab, with more plaque being cleared than the 33% that has been estimated to be directly bound by donanemab [37].

While microglia are accepted to play critical roles in antibody‐dependent plaque clearance, the mechanistic details underlying this are still being explored. Recent studies have demonstrated that engagement of microglial FcRs by anti‐amyloid mAbs is necessary for plaque clearance and suggested microglial pathways that connect antibody action to phagocytosis [38]. As this literature continues to develop, it may be of interest to incorporate the mechanisms uncovered into future QSP models.

The small amount of amyloid plaque bound by donanemab also raises the question of how higher‐order amyloid aggregates can be represented in a biologically realistic way. Across the seven included QSP models, we observed variation in how much of the amyloid within each aggregate is accessible for antibody binding, with a number of models considering all amyloid within each pool to be available. Model 6 was the only model to simulate donanemab. Donanemab's different mechanism of action, binding pyroglutamate modified Aβ specifically, was captured through the plaque‐affinity parameter only, without restricting amyloid availability by explicitly modeling the modification.

In addition to the existence of specific modifications, amyloid plaques have complex internal structures and there is variability across the brain in terms of plaque number, size, and maturity. Although incorporating such details would increase model complexity and therefore should be considered within the context of the model's purpose, these factors do influence the model predicted amyloid:antibody brain ratio. Recent results for trontinemab suggest that the higher antibody:amyloid brain ratio achieved by this drug is a key driving force behind its improved ability to remove plaque from the brain [39].

Understanding the effect of simplifying representations of plaque and plaque clearance to different degrees will be important for guiding further model development. The limited information on model development and use provided in these publications and the absence of model code meant that model reproduction was not feasible. At this stage, it is therefore not possible to provide further insight into this question.

### 4.4 Limited Reproducibility Across QSP Models

Our assessment of model quality revealed a frequent lack of methodological details as well as the absence of executable code across all models included in the review. This information is essential to allow for proper model reproduction, which in turn would allow for better interrogation of models leading to improved suggestions for model development.

Absences of code have been identified previously. In one study looking at the reproducibility of QSP models, of the 12 models assessed for code reproducibility, only four provided readily executable scripts relating to the simulations presented [13]. The previous scoping review on mathematical models of AD also found a clear minority of models (1/17) published their code [18], as did a review of neurodegenerative disease QSP models generally, where code was publicly available for 7/33 models [40]. In addition, publishing code alone is not necessarily sufficient for reproducibility—it should also be properly documented to allow proper use of the model by others.

Further, while model equations and parameters were provided for all models considered, meaning the models could in theory be implemented, the lack of information surrounding model solving and model inputs means in practice it is not guaranteed specific results could be replicated. In this context, the provision of executable code is a valuable benchmark for reproducibility.

Issues with reproducibility are also related to the observations that highly similar models are frequently developed de novo rather than using or extending previously published models [12, 41]. The structural similarity of the six independently developed models analyzed in this review has been highlighted above. The similarity was so strong that in some cases parameter values could be directly compared.

There is of course a balance to be struck between the benefits of independent model development in demonstrating the robustness of the approach and catching errors on the one hand, versus the cost of wasted time on the other. However, better explanation and justification of modeling choices and assumptions make it easier to assess existing models for their suitability to new questions of interest, and therefore to evaluate whether reuse or extension of a model is appropriate. Frameworks for documenting the rationale underlying models have been presented previously [42]. The need for such justifications is demonstrated by an observation made here, where models 4 and 7 derived their steady‐state brain amyloid concentrations from the same paper, but obtained different values.

### 4.5 Limitations of Review

One limitation of this systematic review, which stems from the variation in terminology used to describe QSP models, is the fact that not all relevant publications will have been identified by our search strategy. Despite efforts to account for synonyms and the use of a broad search strategy, some publications that do not describe the model as “mathematical” or “computational,” at least not in the title or abstract, will not have been identified. As explained above, the initial choices made in defining QSP for the purposes of screening did not always align with the use of the term in the literature. This distinction emphasizes the spectrum of modeling approaches used in practice, as well as the difficulty in assigning definitions to terms and methodologies that are relatively immature and which have not yet been subject to standardization.

A further constraint on the QSP models we were able to identify is the balance between internal and external presentation of models developed in industry. A recent survey of QSP practitioners conducted by the International Consortium for Innovation and Quality in Pharmaceutical Development found that less than 30% of respondents “usually” or “always” presented QSP work at external conferences [43] and 23.2% do not publish their QSP models. This presents challenges in allowing “the community to coalesce around standard procedures”. In a similar vein, this low publication rate also limits the ability of a systematic review such as this to describe the true breadth of approaches.

### 4.6 Opportunities for Future Development

The work done to date in applying QSP models to anti‐amyloid therapeutics for AD is promising and has provided valuable insights into how the pathways underlying AD interface with amyloid targeting therapies. The recent applications of models to questions around maintenance therapies and treatment effects in different populations are two uses that could immediately inform existing and novel AD treatments. However, there is significant potential for further development. Looking forwards, opportunities for QSP exist in the ongoing development of combination therapies and active immunotherapies [44]. In the context of immune‐oncology (IO) modeling, a survey by the IO QSP working group found over 30% of respondents believed QSP could be more frequently applied to clinical trial design and patient stratification [45]—the same could also be said within the AD field.

To fully realize QSP's potential, improving standardization and reproducibility will be paramount. Regulatory bodies, such as the FDA, have a crucial role in shaping standards. The evolution of a risk‐based approach to QSP submissions since 2013 shows progress [9], but a more formal framework, similar to what has been developed for classical PKPD models [11], could be beneficial. Recently, the EMA has widened the scope of its guideline on the assessment and reporting of mechanistic models to include QSP, with an updated guideline reflecting this change expected in 2026 [46]. These and other guidelines, such as those developed by the International Council for Harmonization [47], have focused on standardizing regulatory interactions and reporting. At the same time, it is crucial that the quality‐related concerns identified in this review are addressed, potentially through more technically focused guidelines. The importance of parameter identifiability, model calibration, and validation are discussed above, and should guide both model development and publication. Improving reproducibility in these ways would foster transparency and collaboration, driving the development of more robust and more broadly applicable models.

Requiring the inclusion of code when submitting to journals would greatly support these endeavors. Efforts to develop repositories for mathematical models in general, such as BioModels (https://biomodels.org/), have led to a number of journals recommending model submission alongside article submission [48]. However, the low publication rates of QSP models developed in industry are indicative of IP considerations that may also present challenges in the publication of code. Furthermore, while the publication of code could be incentivized, ensuring reproducibility of that code through good documentation and maintenance practices is a distinct and more challenging issue. Indeed, a study of models deposited to BioModels found that 49% were not directly reproducible [49].

Strong collaborations between academia and industry could help navigate these challenges by promoting the development of open source “platform” models for AD, representing a much wider range of biological pathways [8]. Such models would better capture the biology underlying the disease, allowing us to probe our understanding and more easily allow for simulations of combination therapies. Expanding the scope of QSP models in the direction of tau, neurodegeneration, and immune responses offers promising opportunities for advancing AD models. As evidence for how these other pathways contribute to AD onset and development continues to build, QSP models incorporating these pathological changes could aid the discovery of new therapeutic targets.

Collaboration and feedback between experimentalists and modelers could also play a substantial role in improving the data landscape by promoting data acquisition targeted at existing knowledge gaps identified by clinically relevant modeling. Although the availability of data faces many of the same challenges as the provision of code (only 8% of clinical modelers publish their model alongside all data used to develop the model [43]), it is clear that access to more and better data could enhance future models, as would the development of new tools to integrate more varied sources of data [12, 45].

## 5 Conclusion

The QSP models for AD reviewed here, though all developed recently and often with limited data, have provided insights into several questions central to ongoing clinical development. The UK QSP Network describes QSP as a “new emerging area of science,” indicative of the potential of the approach. Looking towards avenues for future development, the issues with model calibration, validation, and reproducibility identified in this review are areas ripe for improvement. Expanding the scope of amyloid‐focused QSP models in the direction of tau, neurodegeneration, and immune responses also offer opportunities for advancing models for AD. In both cases, regulatory input and the establishment of collaborative model development platforms could help improve reproducibility and data sharing. The QSP models evaluated in this review can serve as stepping stones for future development, being refined and expanded for applications to novel therapies and therapeutic strategies.

## Author Contributions

L.H., M.C., N.F., E.G., and J.W. designed the research. L.H. performed the research and analyzed the data. L.H. wrote the manuscript (original draft) and all authors reviewed and edited the manuscript.

## Funding

This work was supported by funding from the Engineering and Physical Sciences Research Council (EPSRC) (grant number EP/S024093/1).

## Conflicts of Interest

L.H. has received funding from AC Immune SA. N.F. and J.W. are employees of AC Immune SA and also hold shares in AC Immune SA.

## Supporting information


**Data S1:** psp470223‐sup‐0001‐Supinfo.docx.

**Table S1:** Log of all literature screened.

**Table S2:** Inclusion/exclusion details for full‐text screening.

**Table S3:** Summaries of models excluded during full‐text screening with clinically relevant outputs.

**Table S4:** Data extracted from the seven included studies.

**Table S5:** Model quality scores.

**Table S6:** Comparison of antibody affinities to different amyloid species used in modeling.
