# Supplementary material for: Quantitative Systems Pharmacology Models of Anti‐Amyloid Treatments for Alzheimer's Disease: A Systematic Review
Source: CPT Pharmacometrics Syst Pharmacol. 2026 Mar 5;15(3):e70223. doi: 10.1002/psp4.70223 (PMC12962395; doi:10.1002/psp4.70223)
Supplement: Supplementary file 1 — Data S1: psp470223‐sup‐0001‐Supinfo.docx. [file PSP4-15-e70223-s005.docx]

**Supplementary Information for Quantitative Systems Pharmacology Models of Anti-Amyloid Treatments for Alzheimer’s Disease: A Systematic Review**

[*S1.* *Search terms* 2](#_Toc201850745)

[S1.1. Scopus (Figure 1) 2](#_Toc201850746)

[S1.2. PubMed 2](#_Toc201850747)

[S1.3. Web of Science 13](#_Toc201850748)

[S2. *Complete screening summary statistics* 19](#_Toc201850749)

[S3. *Summaries of the seven included anti-amyloid QSP models* 20](#_Toc201850750)

[S4. *Conversion of fractional rates* 21](#_Toc201850751)

[S5. *List of Supplementary Tables* 22](#_Toc201850752)

[*References* 23](#_Toc201850753)

# *Search terms*

## Scopus (Figure 1)

TITLE-ABS-KEY(``quantitative systems pharmacology") (blue); of which TITLE-ABS-KEY(``quantitative systems pharmacology" AND (alzheimer* OR neurodegenerat*)) (orange). The search was conducted on 15th October 2024.

## PubMed

(("alzheimer*"[Title] OR "amyloid"[Title] OR "tau"[Title]) AND ("model*"[Title] OR "simulat*"[Title] OR "in silico"[Title] OR "quantitative systems pharmacology"[Title]) AND (("math*"[Title/Abstract] OR "comput*"[Title/Abstract] OR "in silico"[Title/Abstract] OR "quantitative systems pharmacology"[Title/Abstract] OR "qsp"[Title/Abstract] OR "pharmacokinetic"[Title/Abstract] OR "pharmacodynamic"[Title/Abstract]) AND ("antibod*"[Title/Abstract] OR "therap*"[Title/Abstract] OR "drug"[Title/Abstract] OR "intervention*"[Title/Abstract] OR "agent*"[Title/Abstract]))) NOT ( (("animal experimentation"[MeSH Terms] OR "models, animal"[MeSH Terms] OR "animals"[MeSH Terms:noexp] OR "animal population groups"[MeSH Terms] OR "chordata"[MeSH Terms:noexp] OR "vertebrates"[MeSH Terms:noexp] OR "amphibians"[MeSH Terms] OR "birds"[MeSH Terms] OR "fishes"[MeSH Terms] OR "reptiles"[MeSH Terms] OR "mammals"[MeSH Terms:noexp] OR "primates"[MeSH Terms:noexp] OR "eutheria"[MeSH Terms:noexp] OR "artiodactyla"[MeSH Terms] OR "carnivora"[MeSH Terms] OR "cephalopoda"[MeSH Terms] OR "cetacea"[MeSH Terms] OR "chiroptera"[MeSH Terms] OR "elephants"[MeSH Terms] OR "hyraxes"[MeSH Terms] OR "eulipotyphla"[MeSH Terms] OR "lagomorpha"[MeSH Terms] OR "marsupialia"[MeSH Terms] OR "monotremata"[MeSH Terms] OR "perissodactyla"[MeSH Terms] OR "proboscidea mammal"[MeSH Terms:noexp] OR "rodentia"[MeSH Terms] OR "scandentia"[MeSH Terms] OR "sirenia"[MeSH Terms] OR "xenarthra"[MeSH Terms] OR "haplorhini"[MeSH Terms:noexp] OR "strepsirhini"[MeSH Terms] OR "platyrrhini"[MeSH Terms] OR "tarsii"[MeSH Terms] OR "catarrhini"[MeSH Terms:noexp] OR "cercopithecidae"[MeSH Terms] OR "hylobatidae"[MeSH Terms] OR "hominidae"[MeSH Terms:noexp] OR "gorilla gorilla"[MeSH Terms] OR "pan paniscus"[MeSH Terms] OR "pan troglodytes"[MeSH Terms] OR "pongo"[MeSH Terms] OR "rat"[Title/Abstract] OR "rats"[Title/Abstract] OR "animal"[Title/Abstract] OR "animals"[Title/Abstract] OR "mice"[Title/Abstract] OR "mouse"[Title/Abstract] OR "rabbit"[Title/Abstract] OR "rabbits"[Title/Abstract] OR "murine"[Title/Abstract] OR "pig"[Title/Abstract] OR "pigs"[Title/Abstract] OR "dog"[Title/Abstract] OR "dogs"[Title/Abstract] OR "bovine"[Title/Abstract] OR "fish"[Title/Abstract] OR "vertebrate"[Title/Abstract] OR "vertebrates"[Title/Abstract] OR "cat"[Title/Abstract] OR "cats"[Title/Abstract] OR "rodent"[Title/Abstract] OR "rodents"[Title/Abstract] OR "mammal"[Title/Abstract] OR "mammals"[Title/Abstract] OR "chicken"[Title/Abstract] OR "chickens"[Title/Abstract] OR "monkey"[Title/Abstract] OR "monkeys"[Title/Abstract] OR "sheep"[Title/Abstract] OR "canine"[Title/Abstract] OR "canines"[Title/Abstract] OR "porcine"[Title/Abstract] OR "cattle"[Title/Abstract] OR "bird"[Title/Abstract] OR "birds"[Title/Abstract] OR "hamster"[Title/Abstract] OR "hamsters"[Title/Abstract] OR "primate"[Title/Abstract] OR "primates"[Title/Abstract] OR "cow"[Title/Abstract] OR "cows"[Title/Abstract] OR "chick"[Title/Abstract] OR "horse"[Title/Abstract] OR "horses"[Title/Abstract] OR "avian"[Title/Abstract] OR "avians"[Title/Abstract] OR "calf"[Title/Abstract] OR "swine"[Title/Abstract] OR "swines"[Title/Abstract] OR "xenopus"[Title/Abstract] OR "turkeys"[Title/Abstract] OR "bear"[Title/Abstract] OR "bears"[Title/Abstract] OR "frog"[Title/Abstract] OR "frogs"[Title/Abstract] OR "zebrafish"[Title/Abstract] OR "goat"[Title/Abstract] OR "goats"[Title/Abstract] OR "equine"[Title/Abstract] OR "calves"[Title/Abstract] OR "poultry"[Title/Abstract] OR "macaque"[Title/Abstract] OR "macaques"[Title/Abstract] OR "mole"[Title/Abstract] OR "moles"[Title/Abstract] OR "ovine"[Title/Abstract] OR "lamb"[Title/Abstract] OR "lambs"[Title/Abstract] OR "fishes"[Title/Abstract] OR "diptera"[Title/Abstract] OR "amphibian"[Title/Abstract] OR "amphibians"[Title/Abstract] OR "snake"[Title/Abstract] OR "snakes"[Title/Abstract] OR "ruminant"[Title/Abstract] OR "ruminants"[Title/Abstract] OR "hen"[Title/Abstract] OR "hens"[Title/Abstract] OR "piglet"[Title/Abstract] OR "piglets"[Title/Abstract] OR "feline"[Title/Abstract] OR "felines"[Title/Abstract] OR "simian"[Title/Abstract] OR "simians"[Title/Abstract] OR "laevis"[Title/Abstract] OR "trout"[Title/Abstract] OR "trouts"[Title/Abstract] OR "teleost"[Title/Abstract] OR "teleosts"[Title/Abstract] OR "salmon"[Title/Abstract] OR "salmons"[Title/Abstract] OR "seal"[Title/Abstract] OR "seals"[Title/Abstract] OR "bull"[Title/Abstract] OR "bulls"[Title/Abstract] OR "ewe"[Title/Abstract] OR "ewes"[Title/Abstract] OR "hedgehog"[Title/Abstract] OR "hedgehogs"[Title/Abstract] OR "macaca"[Title/Abstract] OR "macacas"[Title/Abstract] OR "proteus"[Title/Abstract] OR "pigeon"[Title/Abstract] OR "pigeons"[Title/Abstract] OR "bat"[Title/Abstract] OR "bats"[Title/Abstract] OR "duck"[Title/Abstract] OR "ducks"[Title/Abstract] OR "chimpanzee"[Title/Abstract] OR "chimpanzees"[Title/Abstract] OR "baboon"[Title/Abstract] OR "baboons"[Title/Abstract] OR "deer"[Title/Abstract] OR "rana"[Title/Abstract] OR "ranas"[Title/Abstract] OR "carp"[Title/Abstract] OR "carps"[Title/Abstract] OR "heifer"[Title/Abstract] OR "swallow"[Title/Abstract] OR "swallows"[Title/Abstract] OR "lizard"[Title/Abstract] OR "lizards"[Title/Abstract] OR "canis"[Title/Abstract] OR "sow"[Title/Abstract] OR "sows"[Title/Abstract] OR "cynomolgus"[Title/Abstract] OR "quail"[Title/Abstract] OR "quails"[Title/Abstract] OR "reptile"[Title/Abstract] OR "reptiles"[Title/Abstract] OR "turtle"[Title/Abstract] OR "turtles"[Title/Abstract] OR "buffalo"[Title/Abstract] OR "gerbil"[Title/Abstract] OR "gerbils"[Title/Abstract] OR "boar"[Title/Abstract] OR "boars"[Title/Abstract] OR "squirrel"[Title/Abstract] OR "squirrels"[Title/Abstract] OR "oncorhynchus"[Title/Abstract] OR "mus"[Title/Abstract] OR "toad"[Title/Abstract] OR "toads"[Title/Abstract] OR "fowl"[Title/Abstract] OR "fowls"[Title/Abstract] OR "rerio"[Title/Abstract] OR "danio"[Title/Abstract] OR "ara"[Title/Abstract] OR "aras"[Title/Abstract] OR "musculus"[Title/Abstract] OR "tadpole"[Title/Abstract] OR "tadpoles"[Title/Abstract] OR "mulatta"[Title/Abstract] OR "salmo"[Title/Abstract] OR "ram"[Title/Abstract] OR "eagle"[Title/Abstract] OR "eagles"[Title/Abstract] OR "ferret"[Title/Abstract] OR "ferrets"[Title/Abstract] OR "goldfish"[Title/Abstract] OR "catfish"[Title/Abstract] OR "whale"[Title/Abstract] OR "whales"[Title/Abstract] OR "fox"[Title/Abstract] OR "foxes"[Title/Abstract] OR "ape"[Title/Abstract] OR "apes"[Title/Abstract] OR "elephant"[Title/Abstract] OR "elephants"[Title/Abstract] OR "bos"[Title/Abstract] OR "marmoset"[Title/Abstract] OR "marmosets"[Title/Abstract] OR "cod"[Title/Abstract] OR "cods"[Title/Abstract] OR "shark"[Title/Abstract] OR "sharks"[Title/Abstract] OR "wolf"[Title/Abstract] OR "eel"[Title/Abstract] OR "eels"[Title/Abstract] OR "auratus"[Title/Abstract] OR "rattus"[Title/Abstract] OR "zebra"[Title/Abstract] OR "zebras"[Title/Abstract] OR "tilapia"[Title/Abstract] OR "tilapias"[Title/Abstract] OR "gilt"[Title/Abstract] OR "camel"[Title/Abstract] OR "camels"[Title/Abstract] OR "squid"[Title/Abstract] OR "gallus"[Title/Abstract] OR "marsupial"[Title/Abstract] OR "marsupials"[Title/Abstract] OR "vole"[Title/Abstract] OR "voles"[Title/Abstract] OR "fascicularis"[Title/Abstract] OR "ovis"[Title/Abstract] OR "salmonid"[Title/Abstract] OR "salmonids"[Title/Abstract] OR "tiger"[Title/Abstract] OR "tigers"[Title/Abstract] OR "dolphin"[Title/Abstract] OR "dolphins"[Title/Abstract] OR "robin"[Title/Abstract] OR "robins"[Title/Abstract] OR "carpio"[Title/Abstract] OR "opossum"[Title/Abstract] OR "opossums"[Title/Abstract] OR "cyprinus"[Title/Abstract] OR "salamander"[Title/Abstract] OR "salamanders"[Title/Abstract] OR "felis"[Title/Abstract] OR "mink"[Title/Abstract] OR "minks"[Title/Abstract] OR "swan"[Title/Abstract] OR "swans"[Title/Abstract] OR "norvegicus"[Title/Abstract] OR "bufo"[Title/Abstract] OR "torpedo"[Title/Abstract] OR "bass"[Title/Abstract] OR "lamprey"[Title/Abstract] OR "lampreys"[Title/Abstract] OR "sus"[Title/Abstract] OR "python"[Title/Abstract] OR "pythons"[Title/Abstract] OR "tetrapod"[Title/Abstract] OR "tetrapods"[Title/Abstract] OR "shrew"[Title/Abstract] OR "shrews"[Title/Abstract] OR "lion"[Title/Abstract] OR "lions"[Title/Abstract] OR "hog"[Title/Abstract] OR "hogs"[Title/Abstract] OR "songbird"[Title/Abstract] OR "songbirds"[Title/Abstract] OR "oreochromis"[Title/Abstract] OR "starling"[Title/Abstract] OR "starlings"[Title/Abstract] OR "caprine"[Title/Abstract] OR "carassius"[Title/Abstract] OR "owl"[Title/Abstract] OR "owls"[Title/Abstract] OR "newt"[Title/Abstract] OR "newts"[Title/Abstract] OR "papio"[Title/Abstract] OR "scrofa"[Title/Abstract] OR "hare"[Title/Abstract] OR "hares"[Title/Abstract] OR "gorilla"[Title/Abstract] OR "gorillas"[Title/Abstract] OR "flounder"[Title/Abstract] OR "flounders"[Title/Abstract] OR "goose"[Title/Abstract] OR "herring"[Title/Abstract] OR "herrings"[Title/Abstract] OR "therian"[Title/Abstract] OR "buffaloes"[Title/Abstract] OR "canary"[Title/Abstract] OR "sparrow"[Title/Abstract] OR "sparrows"[Title/Abstract] OR "microtus"[Title/Abstract] OR "octopus"[Title/Abstract] OR "troglodytes"[Title/Abstract] OR "tuna"[Title/Abstract] OR "amphibia"[Title/Abstract] OR "chinchilla"[Title/Abstract] OR "chinchillas"[Title/Abstract] OR "ide"[Title/Abstract] OR "oryzias"[Title/Abstract] OR "cervus"[Title/Abstract] OR "kangaroo"[Title/Abstract] OR "kangaroos"[Title/Abstract] OR "armadillo"[Title/Abstract] OR "armadillos"[Title/Abstract] OR "callithrix"[Title/Abstract] OR "pan troglodytes"[Title/Abstract] OR "saimiri"[Title/Abstract] OR "cichlid"[Title/Abstract] OR "cichlids"[Title/Abstract] OR "donkey"[Title/Abstract] OR "donkeys"[Title/Abstract] OR "bream"[Title/Abstract] OR "char"[Title/Abstract] OR "chars"[Title/Abstract] OR "finch"[Title/Abstract] OR "raccoon"[Title/Abstract] OR "raccoons"[Title/Abstract] OR "bothrops"[Title/Abstract] OR "anguilla"[Title/Abstract] OR "perch"[Title/Abstract] OR "cricetus"[Title/Abstract] OR "seabird"[Title/Abstract] OR "seabirds"[Title/Abstract] OR "buck"[Title/Abstract] OR "bucks"[Title/Abstract] OR "naja"[Title/Abstract] OR "coturnix"[Title/Abstract] OR "salmonids"[Title/Abstract] OR "geese"[Title/Abstract] OR "minnow"[Title/Abstract] OR "minnows"[Title/Abstract] OR "raptor"[Title/Abstract] OR "raptors"[Title/Abstract] OR "merione"[Title/Abstract] OR "meriones"[Title/Abstract] OR "rodentia"[Title/Abstract] OR "elaphus"[Title/Abstract] OR "amniote"[Title/Abstract] OR "amniotes"[Title/Abstract] OR "elasmobranch"[Title/Abstract] OR "emu"[Title/Abstract] OR "emus"[Title/Abstract] OR "peromyscus"[Title/Abstract] OR "hominid"[Title/Abstract] OR "hominids"[Title/Abstract] OR "bubalus"[Title/Abstract] OR "crotalus"[Title/Abstract] OR "gull"[Title/Abstract] OR "gulls"[Title/Abstract] OR "anas"[Title/Abstract] OR "anura"[Title/Abstract] OR "lemur"[Title/Abstract] OR "lemurs"[Title/Abstract] OR "crow"[Title/Abstract] OR "crows"[Title/Abstract] OR "camelus"[Title/Abstract] OR "gibbon"[Title/Abstract] OR "gibbons"[Title/Abstract] OR "waterfowl"[Title/Abstract] OR "parrot"[Title/Abstract] OR "parrots"[Title/Abstract] OR "eels"[Title/Abstract] OR "cob"[Title/Abstract] OR "stickleback"[Title/Abstract] OR "sticklebacks"[Title/Abstract] OR "columba"[Title/Abstract] OR "mesocricetus"[Title/Abstract] OR "ambystoma"[Title/Abstract] OR "raven"[Title/Abstract] OR "ravens"[Title/Abstract] OR "gadus"[Title/Abstract] OR "penguin"[Title/Abstract] OR "penguins"[Title/Abstract] OR "orangutan"[Title/Abstract] OR "orangutans"[Title/Abstract] OR "sturgeon"[Title/Abstract] OR "sturgeons"[Title/Abstract] OR "cuniculus"[Title/Abstract] OR "aves"[Title/Abstract] OR "virginianus"[Title/Abstract] OR "cephalopod"[Title/Abstract] OR "cephalopods"[Title/Abstract] OR "cebus"[Title/Abstract] OR "sparus"[Title/Abstract] OR "tortoise"[Title/Abstract] OR "tortoises"[Title/Abstract] OR "guttata"[Title/Abstract] OR "morhua"[Title/Abstract] OR "unguiculatus"[Title/Abstract] OR "dogfish"[Title/Abstract] OR "vulpes"[Title/Abstract] OR "mallard"[Title/Abstract] OR "mallards"[Title/Abstract] OR "apodemus"[Title/Abstract] OR "alligator"[Title/Abstract] OR "alligators"[Title/Abstract] OR "oryctolagus"[Title/Abstract] OR "llama"[Title/Abstract] OR "llamas"[Title/Abstract] OR "reindeer"[Title/Abstract] OR "mustela"[Title/Abstract] OR "duckling"[Title/Abstract] OR "ducklings"[Title/Abstract] OR "wolves"[Title/Abstract] OR "sander"[Title/Abstract] OR "amazona"[Title/Abstract] OR "zebu"[Title/Abstract] OR "badger"[Title/Abstract] OR "badgers"[Title/Abstract] OR "dove"[Title/Abstract] OR "doves"[Title/Abstract] OR "ictalurus"[Title/Abstract] OR "capra"[Title/Abstract] OR "capras"[Title/Abstract] OR "equus"[Title/Abstract] OR "camelid"[Title/Abstract] OR "camelids"[Title/Abstract] OR "poecilia"[Title/Abstract] OR "mule"[Title/Abstract] OR "mules"[Title/Abstract] OR "perciformes"[Title/Abstract] OR "salvelinus"[Title/Abstract] OR "labrax"[Title/Abstract] OR "cyprinidae"[Title/Abstract] OR "ariidae"[Title/Abstract] OR "crocodile"[Title/Abstract] OR "crocodiles"[Title/Abstract] OR "fundulus"[Title/Abstract] OR "dicentrarchus"[Title/Abstract] OR "clarias"[Title/Abstract] OR "cercopithecus"[Title/Abstract] OR "chiroptera"[Title/Abstract] OR "alpaca"[Title/Abstract] OR "alpacas"[Title/Abstract] OR "pike"[Title/Abstract] OR "pikes"[Title/Abstract] OR "paralichthys"[Title/Abstract] OR "puma"[Title/Abstract] OR "pumas"[Title/Abstract] OR "didelphis"[Title/Abstract] OR "pisces"[Title/Abstract] OR "macropus"[Title/Abstract] OR "triturus"[Title/Abstract] OR "bison"[Title/Abstract] OR "bisons"[Title/Abstract] OR "epinephelus"[Title/Abstract] OR "gasterosteus"[Title/Abstract] OR "panthera"[Title/Abstract] OR "acipenser"[Title/Abstract] OR "mackerel"[Title/Abstract] OR "mackerels"[Title/Abstract] OR "tamarin"[Title/Abstract] OR "tamarins"[Title/Abstract] OR "ostrich"[Title/Abstract] OR "anolis"[Title/Abstract] OR "vervet"[Title/Abstract] OR "vervets"[Title/Abstract] OR "wallaby"[Title/Abstract] OR "glareolus"[Title/Abstract] OR "beaver"[Title/Abstract] OR "beavers"[Title/Abstract] OR "dromedary"[Title/Abstract] OR "catus"[Title/Abstract] OR "killifish"[Title/Abstract] OR "pimephales"[Title/Abstract] OR "promelas"[Title/Abstract] OR "aotus"[Title/Abstract] OR "phoca"[Title/Abstract] OR "panda"[Title/Abstract] OR "pandas"[Title/Abstract] OR "porpoise"[Title/Abstract] OR "porpoises"[Title/Abstract] OR "myotis"[Title/Abstract] OR "yak"[Title/Abstract] OR "yaks"[Title/Abstract] OR "agkistrodon"[Title/Abstract] OR "vipera"[Title/Abstract] OR "otter"[Title/Abstract] OR "otters"[Title/Abstract] OR "turbot"[Title/Abstract] OR "turbots"[Title/Abstract] OR "squamate"[Title/Abstract] OR "carnivora"[Title/Abstract] OR "mullet"[Title/Abstract] OR "mullets"[Title/Abstract] OR "hawk"[Title/Abstract] OR "hawks"[Title/Abstract] OR "taeniopygia"[Title/Abstract] OR "seahorse"[Title/Abstract] OR "seahorses"[Title/Abstract] OR "poecilia reticulata"[Title/Abstract] OR "falcon"[Title/Abstract] OR "falcons"[Title/Abstract] OR "prosimian"[Title/Abstract] OR "prosimians"[Title/Abstract] OR "parus"[Title/Abstract] OR "perca"[Title/Abstract] OR "fingerling"[Title/Abstract] OR "fingerlings"[Title/Abstract] OR "antelope"[Title/Abstract] OR "antelopes"[Title/Abstract] OR "tupaia"[Title/Abstract] OR "passeriformes"[Title/Abstract] OR "sepia"[Title/Abstract] OR "saguinus"[Title/Abstract] OR "coyote"[Title/Abstract] OR "coyotes"[Title/Abstract] OR "pongo"[Title/Abstract] OR "meleagris"[Title/Abstract] OR "reptilia"[Title/Abstract] OR "lepus"[Title/Abstract] OR "psittacine"[Title/Abstract] OR "hagfish"[Title/Abstract] OR "warbler"[Title/Abstract] OR "warblers"[Title/Abstract] OR "russell s viper"[Title/Abstract] OR "russell s vipers"[Title/Abstract] OR "smolt"[Title/Abstract] OR "smolts"[Title/Abstract] OR "budgerigar"[Title/Abstract] OR "sardine"[Title/Abstract] OR "sardines"[Title/Abstract] OR "cavia"[Title/Abstract] OR "hyla"[Title/Abstract] OR "pleurodeles"[Title/Abstract] OR "siluriformes"[Title/Abstract] OR "great tit"[Title/Abstract] OR "great tits"[Title/Abstract] OR "guppy"[Title/Abstract] OR "bonobo"[Title/Abstract] OR "bonobos"[Title/Abstract] OR "rutilus"[Title/Abstract] OR "trichosurus"[Title/Abstract] OR "muridae"[Title/Abstract] OR "phodopus"[Title/Abstract] OR "channa"[Title/Abstract] OR "squalus"[Title/Abstract] OR "lynx"[Title/Abstract] OR "sturnus"[Title/Abstract] OR "petromyzon"[Title/Abstract] OR "vitulina"[Title/Abstract] OR "monodelphis"[Title/Abstract] OR "cuttlefish"[Title/Abstract] OR "adder"[Title/Abstract] OR "adders"[Title/Abstract] OR "lepomis"[Title/Abstract] OR "canaria"[Title/Abstract] OR "gambusia"[Title/Abstract] OR "guppies"[Title/Abstract] OR "xiphophorus"[Title/Abstract] OR "flatfish"[Title/Abstract] OR "koala"[Title/Abstract] OR "koalas"[Title/Abstract] OR "labeo"[Title/Abstract] OR "stingray"[Title/Abstract] OR "stingrays"[Title/Abstract] OR "chelonia"[Title/Abstract] OR "lampetra"[Title/Abstract] OR "spermophilus"[Title/Abstract] OR "crocodilian"[Title/Abstract] OR "passer domesticus"[Title/Abstract] OR "sciurus"[Title/Abstract] OR "artiodactyla"[Title/Abstract] OR "ranidae"[Title/Abstract] OR "corvus"[Title/Abstract] OR "necturus"[Title/Abstract] OR "platypus"[Title/Abstract] OR "canaries"[Title/Abstract] OR "bovid"[Title/Abstract] OR "lagopus"[Title/Abstract] OR "trimeresurus"[Title/Abstract] OR "gariepinus"[Title/Abstract] OR "marten"[Title/Abstract] OR "martens"[Title/Abstract] OR "drosophilidae"[Title/Abstract] OR "mugil"[Title/Abstract] OR "sunfish"[Title/Abstract] OR "porcellus"[Title/Abstract] OR "cypriniformes"[Title/Abstract] OR "alouatta"[Title/Abstract] OR "scophthalmus"[Title/Abstract] OR "anser"[Title/Abstract] OR "electrophorus"[Title/Abstract] OR "putorius"[Title/Abstract] OR "iguana"[Title/Abstract] OR "iguanas"[Title/Abstract] OR "lama"[Title/Abstract] OR "lamas"[Title/Abstract] OR "takifugu"[Title/Abstract] OR "circus"[Title/Abstract] OR "eptesicus"[Title/Abstract] OR "flycatcher"[Title/Abstract] OR "galago"[Title/Abstract] OR "galagos"[Title/Abstract] OR "trachemys"[Title/Abstract] OR "lungfish"[Title/Abstract] OR "characiformes"[Title/Abstract] OR "shorebird"[Title/Abstract] OR "shorebirds"[Title/Abstract] OR "giraffe"[Title/Abstract] OR "giraffes"[Title/Abstract] OR "micropterus"[Title/Abstract] OR "scyliorhinus"[Title/Abstract] OR "cichlidae"[Title/Abstract] OR "loligo"[Title/Abstract] OR "porcupine"[Title/Abstract] OR "porcupines"[Title/Abstract] OR "chub"[Title/Abstract] OR "chubs"[Title/Abstract] OR "solea"[Title/Abstract] OR "pleuronectes"[Title/Abstract] OR "hylidae"[Title/Abstract] OR "viperidae"[Title/Abstract] OR "echis"[Title/Abstract] OR "sorex"[Title/Abstract] OR "anchovy"[Title/Abstract] OR "lagomorph"[Title/Abstract] OR "ostriches"[Title/Abstract] OR "vulture"[Title/Abstract] OR "vultures"[Title/Abstract] OR "whitefish"[Title/Abstract] OR "araneus"[Title/Abstract] OR "jird"[Title/Abstract] OR "jirds"[Title/Abstract] OR "tern"[Title/Abstract] OR "esox"[Title/Abstract] OR "drake"[Title/Abstract] OR "drakes"[Title/Abstract] OR "elapidae"[Title/Abstract] OR "gallopavo"[Title/Abstract] OR "chordata"[Title/Abstract] OR "myodes"[Title/Abstract] OR "caretta"[Title/Abstract] OR "serinus"[Title/Abstract] OR "grouse"[Title/Abstract] OR "misgurnus"[Title/Abstract] OR "meles"[Title/Abstract] OR "blackbird"[Title/Abstract] OR "blackbirds"[Title/Abstract] OR "coregonus"[Title/Abstract] OR "bobwhite"[Title/Abstract] OR "bobwhites"[Title/Abstract] OR "heteropneustes"[Title/Abstract] OR "mammoth"[Title/Abstract] OR "mammoths"[Title/Abstract] OR "turdus"[Title/Abstract] OR "rhinella"[Title/Abstract] OR "ateles"[Title/Abstract] OR "characidae"[Title/Abstract] OR "clupea"[Title/Abstract] OR "bungarus"[Title/Abstract] OR "brill"[Title/Abstract] OR "struthio camelus"[Title/Abstract] OR "sloth"[Title/Abstract] OR "sloths"[Title/Abstract] OR "pteropus"[Title/Abstract] OR "sculpin"[Title/Abstract] OR "anthropoids"[Title/Abstract] OR "pollock"[Title/Abstract] OR "pollocks"[Title/Abstract] OR "morone"[Title/Abstract] OR "pan paniscus"[Title/Abstract] OR "litoria"[Title/Abstract] OR "chipmunk"[Title/Abstract] OR "chipmunks"[Title/Abstract] OR "balaenoptera"[Title/Abstract] OR "marmota"[Title/Abstract] OR "melopsittacus"[Title/Abstract] OR "hyrax"[Title/Abstract] OR "lemming"[Title/Abstract] OR "lemmings"[Title/Abstract] OR "halibut"[Title/Abstract] OR "hylobates"[Title/Abstract] OR "lates"[Title/Abstract] OR "caiman"[Title/Abstract] OR "caimans"[Title/Abstract] OR "sigmodon"[Title/Abstract] OR "stenella"[Title/Abstract] OR "barbel"[Title/Abstract] OR "barbels"[Title/Abstract] OR "sterna"[Title/Abstract] OR "parakeet"[Title/Abstract] OR "parakeets"[Title/Abstract] OR "phocoena"[Title/Abstract] OR "leptodactylus"[Title/Abstract] OR "canidae"[Title/Abstract] OR "buteo"[Title/Abstract] OR "harengus"[Title/Abstract] OR "gopher"[Title/Abstract] OR "gophers"[Title/Abstract] OR "marmot"[Title/Abstract] OR "marmots"[Title/Abstract] OR "gosling"[Title/Abstract] OR "goslings"[Title/Abstract] OR "platichthys"[Title/Abstract] OR "gar"[Title/Abstract] OR "gars"[Title/Abstract] OR "sebastes"[Title/Abstract] OR "marsupialia"[Title/Abstract] OR "notophthalmus"[Title/Abstract] OR "gazelle"[Title/Abstract] OR "gazelles"[Title/Abstract] OR "insectivora"[Title/Abstract] OR "paridae"[Title/Abstract] OR "felidae"[Title/Abstract] OR "russula"[Title/Abstract] OR "galliformes"[Title/Abstract] OR "bombina"[Title/Abstract] OR "colobus"[Title/Abstract] OR "echidna"[Title/Abstract] OR "echidnas"[Title/Abstract] OR "seabass"[Title/Abstract] OR "syncerus"[Title/Abstract] OR "plaice"[Title/Abstract] OR "blue tit"[Title/Abstract] OR "blue tits"[Title/Abstract] OR "pagrus"[Title/Abstract] OR "catfishes"[Title/Abstract] OR "cetacea"[Title/Abstract] OR "barbus"[Title/Abstract] OR "cygnus"[Title/Abstract] OR "ficedula"[Title/Abstract] OR "chamois"[Title/Abstract] OR "colubridae"[Title/Abstract] OR "perches"[Title/Abstract] OR "coelacanth"[Title/Abstract] OR "fitch"[Title/Abstract] OR "urodela"[Title/Abstract] OR "cynops"[Title/Abstract] OR "martes"[Title/Abstract] OR "halichoerus"[Title/Abstract] OR "aix"[Title/Abstract] OR "salmonidae"[Title/Abstract] OR "leuciscus"[Title/Abstract] OR "magpie"[Title/Abstract] OR "magpies"[Title/Abstract] OR "silurus"[Title/Abstract] OR "whiting"[Title/Abstract] OR "whitings"[Title/Abstract] OR "anseriformes"[Title/Abstract] OR "colinus"[Title/Abstract] OR "rhea"[Title/Abstract] OR "chlorocebus"[Title/Abstract] OR "octodon"[Title/Abstract] OR "acinonyx"[Title/Abstract] OR "mouflon"[Title/Abstract] OR "mouflons"[Title/Abstract] OR "ibex"[Title/Abstract] OR "tetraodon"[Title/Abstract] OR "bufonidae"[Title/Abstract] OR "equidae"[Title/Abstract] OR "jackal"[Title/Abstract] OR "cephalopoda"[Title/Abstract] OR "dendroaspis"[Title/Abstract] OR "glama"[Title/Abstract] OR "muskrat"[Title/Abstract] OR "muskrats"[Title/Abstract] OR "sable"[Title/Abstract] OR "sables"[Title/Abstract] OR "wildebeest"[Title/Abstract] OR "streptopelia"[Title/Abstract] OR "albifrons"[Title/Abstract] OR "vespertilionidae"[Title/Abstract] OR "woodpecker"[Title/Abstract] OR "woodpeckers"[Title/Abstract] OR "muntjac"[Title/Abstract] OR "muntjacs"[Title/Abstract] OR "archosaur"[Title/Abstract] OR "branta"[Title/Abstract] OR "cricetulus"[Title/Abstract] OR "megalobrama"[Title/Abstract] OR "poeciliidae"[Title/Abstract] OR "desmodus"[Title/Abstract] OR "snakehead"[Title/Abstract] OR "snakeheads"[Title/Abstract] OR "tench"[Title/Abstract] OR "teal"[Title/Abstract] OR "teals"[Title/Abstract] OR "bandicoot"[Title/Abstract] OR "bandicoots"[Title/Abstract] OR "apteronotus"[Title/Abstract] OR "phyllostomidae"[Title/Abstract] OR "crocidura"[Title/Abstract] OR "buzzard"[Title/Abstract] OR "buzzards"[Title/Abstract] OR "larimichthys"[Title/Abstract] OR "cercocebus"[Title/Abstract] OR "pipistrellus"[Title/Abstract] OR "erithacus"[Title/Abstract] OR "impala"[Title/Abstract] OR "impalas"[Title/Abstract] OR "rousettus"[Title/Abstract] OR "haddock"[Title/Abstract] OR "haddocks"[Title/Abstract] OR "tinca"[Title/Abstract] OR "ratite"[Title/Abstract] OR "calidris"[Title/Abstract] OR "cynoglossus"[Title/Abstract] OR "hypophthalmichthys"[Title/Abstract] OR "bullock"[Title/Abstract] OR "bullocks"[Title/Abstract] OR "dromedaries"[Title/Abstract] OR "alectoris"[Title/Abstract] OR "filly"[Title/Abstract] OR "salamandra"[Title/Abstract] OR "cingulata"[Title/Abstract] OR "bitis"[Title/Abstract] OR "grus"[Title/Abstract] OR "ammodytes"[Title/Abstract] OR "macaw"[Title/Abstract] OR "macaws"[Title/Abstract] OR "hypoleuca"[Title/Abstract] OR "sapajus"[Title/Abstract] OR "cyprinodontiformes"[Title/Abstract] OR "hippopotamus"[Title/Abstract] OR "pelophylax"[Title/Abstract] OR "capybara"[Title/Abstract] OR "capybaras"[Title/Abstract] OR "weasel"[Title/Abstract] OR "weasels"[Title/Abstract] OR "cairina"[Title/Abstract] OR "cynomys"[Title/Abstract] OR "lutra"[Title/Abstract] OR "cockatoo"[Title/Abstract] OR "cockatoos"[Title/Abstract] OR "lachesis"[Title/Abstract] OR "lagomorpha"[Title/Abstract] OR "rupicapra"[Title/Abstract] OR "daboia"[Title/Abstract] OR "orang utan"[Title/Abstract] OR "orang utans"[Title/Abstract] OR "platyrrhini"[Title/Abstract] OR "charadriiformes"[Title/Abstract] OR "micrurus"[Title/Abstract] OR "psittaciformes"[Title/Abstract] OR "spalax"[Title/Abstract] OR "loris"[Title/Abstract] OR "mustelidae"[Title/Abstract] OR "sylvilagus"[Title/Abstract] OR "vitticeps"[Title/Abstract] OR "cockatiel"[Title/Abstract] OR "mustelus"[Title/Abstract] OR "cottus"[Title/Abstract] OR "erythrocebus"[Title/Abstract] OR "dipodomys"[Title/Abstract] OR "platessa"[Title/Abstract] OR "callicebus"[Title/Abstract] OR "loricariidae"[Title/Abstract] OR "catostomus"[Title/Abstract] OR "cuneata"[Title/Abstract] OR "cyanistes"[Title/Abstract] OR "cyprinodon"[Title/Abstract] OR "sigmodontinae"[Title/Abstract] OR "elasmobranchii"[Title/Abstract] OR "trichechus"[Title/Abstract] OR "sauropsid"[Title/Abstract] OR "xenarthra"[Title/Abstract] OR "dormouse"[Title/Abstract] OR "perissodactyla"[Title/Abstract] OR "nautilus"[Title/Abstract] OR "cirrhinus"[Title/Abstract] OR "gulo"[Title/Abstract] OR "tragelaphus"[Title/Abstract] OR "merula"[Title/Abstract] OR "numida"[Title/Abstract] OR "sciaenidae"[Title/Abstract] OR "cerastes"[Title/Abstract] OR "sciuridae"[Title/Abstract] OR "gibbosus"[Title/Abstract] OR "octopuses"[Title/Abstract] OR "eland"[Title/Abstract] OR "elands"[Title/Abstract] OR "phyllomedusa"[Title/Abstract] OR "pogona"[Title/Abstract] OR "walrus"[Title/Abstract] OR "agamidae"[Title/Abstract] OR "leptodactylidae"[Title/Abstract] OR "ridibundus"[Title/Abstract] OR "leontopithecus"[Title/Abstract] OR "anteater"[Title/Abstract] OR "anteaters"[Title/Abstract] OR "pelodiscus"[Title/Abstract] OR "cebidae"[Title/Abstract] OR "columbianus"[Title/Abstract] OR "pelteobagrus fulvidraco"[Title/Abstract] OR "hominoidea"[Title/Abstract] OR "mandrillus"[Title/Abstract] OR "zonotrichia leucophrys"[Title/Abstract] OR "agama"[Title/Abstract] OR "gobiocypris"[Title/Abstract] OR "bearded dragon"[Title/Abstract] OR "bearded dragons"[Title/Abstract] OR "sarotherodon"[Title/Abstract] OR "talpa"[Title/Abstract] OR "discoglossus"[Title/Abstract] OR "hagfishes"[Title/Abstract] OR "sphenodon"[Title/Abstract] OR "gudgeon"[Title/Abstract] OR "amphiuma"[Title/Abstract] OR "aythya"[Title/Abstract] OR "tenrec"[Title/Abstract] OR "tenrec"[Title/Abstract] OR "hominidae"[Title/Abstract] OR "risoria"[Title/Abstract] OR "salamandridae"[Title/Abstract] OR "camelidae"[Title/Abstract] OR "columbiformes"[Title/Abstract] OR "latimeria"[Title/Abstract] OR "plover"[Title/Abstract] OR "plovers"[Title/Abstract] OR "afrotheria"[Title/Abstract] OR "falco sparverius"[Title/Abstract] OR "polecat"[Title/Abstract] OR "polecats"[Title/Abstract] OR "crotalinae"[Title/Abstract] OR "salvadora"[Title/Abstract] OR "tarsier"[Title/Abstract] OR "lucioperca"[Title/Abstract] OR "anchovies"[Title/Abstract] OR "lungfishes"[Title/Abstract] OR "terrapin"[Title/Abstract] OR "dromaius novaehollandiae"[Title/Abstract] OR "lateolabrax"[Title/Abstract] OR "eigenmannia"[Title/Abstract] OR "pelamis"[Title/Abstract] OR "theropithecus"[Title/Abstract] OR "murinae"[Title/Abstract] OR "gander"[Title/Abstract] OR "gymnotus"[Title/Abstract] OR "pseudacris"[Title/Abstract] OR "gymnophiona"[Title/Abstract] OR "gymnotiformes"[Title/Abstract] OR "laticauda"[Title/Abstract] OR "falconiformes"[Title/Abstract] OR "dugong"[Title/Abstract] OR "dugongs"[Title/Abstract] OR "pintail"[Title/Abstract] OR "pintails"[Title/Abstract] OR "rook"[Title/Abstract] OR "rooks"[Title/Abstract] OR "lasiurus"[Title/Abstract] OR "catshark"[Title/Abstract] OR "catsharks"[Title/Abstract] OR "micropogonias"[Title/Abstract] OR "red junglefowl"[Title/Abstract] OR "paddlefish"[Title/Abstract] OR "ophiophagus"[Title/Abstract] OR "hollandicus"[Title/Abstract] OR "nymphicus"[Title/Abstract] OR "pimelodidae"[Title/Abstract] OR "aepyceros"[Title/Abstract] OR "cobitidae"[Title/Abstract] OR "strigiformes"[Title/Abstract] OR "cobitis"[Title/Abstract] OR "dormice"[Title/Abstract] OR "alytes"[Title/Abstract] OR "calloselasma"[Title/Abstract] OR "guanaco"[Title/Abstract] OR "phasianidae"[Title/Abstract] OR "round goby"[Title/Abstract] OR "trichogaster"[Title/Abstract] OR "catarrhini"[Title/Abstract] OR "eelpout"[Title/Abstract] OR "eelpouts"[Title/Abstract] OR "galaxias"[Title/Abstract] OR "gaur"[Title/Abstract] OR "pungitius"[Title/Abstract] OR "suslik"[Title/Abstract] OR "susliks"[Title/Abstract] OR "flatfishes"[Title/Abstract] OR "percidae"[Title/Abstract] OR "caprinae"[Title/Abstract] OR "todarodes"[Title/Abstract] OR "osmerus"[Title/Abstract] OR "ameiurus"[Title/Abstract] OR "anthropoidea"[Title/Abstract] OR "castor canadensis"[Title/Abstract] OR "pouting"[Title/Abstract] OR "tetraodontiformes"[Title/Abstract] OR "arvicolinae"[Title/Abstract] OR "siamang"[Title/Abstract] OR "siamangs"[Title/Abstract] OR "castor fiber"[Title/Abstract] OR "nomascus"[Title/Abstract] OR "red knot"[Title/Abstract] OR "red knots"[Title/Abstract] OR "syngnathidae"[Title/Abstract] OR "iguanidae"[Title/Abstract] OR "eretmochelys"[Title/Abstract] OR "ursidae"[Title/Abstract] OR "callimico"[Title/Abstract] OR "columbidae"[Title/Abstract] OR "microhylidae"[Title/Abstract] OR "anaxyrus"[Title/Abstract] OR "menidia"[Title/Abstract] OR "pipistrelle"[Title/Abstract] OR "greylag"[Title/Abstract] OR "pipidae"[Title/Abstract] OR "scandentia"[Title/Abstract] OR "bowfin"[Title/Abstract] OR "bowfins"[Title/Abstract] OR "dendrobatidae"[Title/Abstract] OR "zenaida"[Title/Abstract] OR "bushbaby"[Title/Abstract] OR "harrier"[Title/Abstract] OR "harriers"[Title/Abstract] OR "macropodidae"[Title/Abstract] OR "pygerythrus"[Title/Abstract] OR "clupeidae"[Title/Abstract] OR "odorrana"[Title/Abstract] OR "corvidae"[Title/Abstract] OR "jerboa"[Title/Abstract] OR "jerboas"[Title/Abstract] OR "canutus"[Title/Abstract] OR "hylobatidae"[Title/Abstract] OR "clupeiformes"[Title/Abstract] OR "great cormorant"[Title/Abstract] OR "great cormorants"[Title/Abstract] OR "scorpaeniformes"[Title/Abstract] OR "chondrostean"[Title/Abstract] OR "garfish"[Title/Abstract] OR "proboscidea"[Title/Abstract] OR "psetta"[Title/Abstract] OR "diapsid"[Title/Abstract] OR "serotinus"[Title/Abstract] OR "tetrao"[Title/Abstract] OR "walruses"[Title/Abstract] OR "carcharhiniformes"[Title/Abstract] OR "leucoraja"[Title/Abstract] OR "pumpkinseed"[Title/Abstract] OR "dosidicus"[Title/Abstract] OR "acipenseriformes"[Title/Abstract] OR "daubentonii"[Title/Abstract] OR "emberizidae"[Title/Abstract] OR "gadiformes"[Title/Abstract] OR "hyraxes"[Title/Abstract] OR "stizostedion"[Title/Abstract] OR "wolverine"[Title/Abstract] OR "wolverines"[Title/Abstract] OR "lissotriton"[Title/Abstract] OR "acanthurus"[Title/Abstract] OR "centrarchidae"[Title/Abstract] OR "gloydius"[Title/Abstract] OR "laurasiatheria"[Title/Abstract] OR "limosa"[Title/Abstract] OR "psittacula"[Title/Abstract] OR "leporidae"[Title/Abstract] OR "proteidae"[Title/Abstract] OR "zander"[Title/Abstract] OR "zanders"[Title/Abstract] OR "arapaima"[Title/Abstract] OR "bagridae"[Title/Abstract] OR "cyprinodontidae"[Title/Abstract] OR "mithun"[Title/Abstract] OR "pandion"[Title/Abstract] OR "jackdaw"[Title/Abstract] OR "jackdaws"[Title/Abstract] OR "procyonidae"[Title/Abstract] OR "carus"[Title/Abstract] OR "jaculus"[Title/Abstract] OR "salmoniformes"[Title/Abstract] OR "common sole"[Title/Abstract] OR "common soles"[Title/Abstract] OR "protobothrops"[Title/Abstract] OR "calamita"[Title/Abstract] OR "brachyteles"[Title/Abstract] OR "trionyx"[Title/Abstract] OR "turdidae"[Title/Abstract] OR "boidae"[Title/Abstract] OR "luscinia"[Title/Abstract] OR "pugnax"[Title/Abstract] OR "euarchontoglires"[Title/Abstract] OR "saithe"[Title/Abstract] OR "saithes"[Title/Abstract] OR "symphalangus"[Title/Abstract] OR "aardvark"[Title/Abstract] OR "aardvarks"[Title/Abstract] OR "oystercatcher"[Title/Abstract] OR "oystercatchers"[Title/Abstract] OR "arius"[Title/Abstract] OR "corydoras"[Title/Abstract] OR "poacher"[Title/Abstract] OR "poachers"[Title/Abstract] OR "aurochs"[Title/Abstract] OR "cebuella"[Title/Abstract] OR "crecca"[Title/Abstract] OR "lemuridae"[Title/Abstract] OR "sirenia"[Title/Abstract] OR "lemmus"[Title/Abstract] OR "perdix"[Title/Abstract] OR "glires"[Title/Abstract] OR "lepidosaur"[Title/Abstract] OR "muskox"[Title/Abstract] OR "deinagkistrodon"[Title/Abstract] OR "pholidota"[Title/Abstract] OR "holocephali"[Title/Abstract] OR "cercopithecinae"[Title/Abstract] OR "clariidae"[Title/Abstract] OR "agapornis"[Title/Abstract] OR "doryteuthis"[Title/Abstract] OR "tyrannidae"[Title/Abstract] OR "dicroglossidae"[Title/Abstract] OR "godwit"[Title/Abstract] OR "godwits"[Title/Abstract] OR "monedula"[Title/Abstract] OR "pongidae"[Title/Abstract] OR "atheriniformes"[Title/Abstract] OR "colobinae"[Title/Abstract] OR "lophocebus"[Title/Abstract] OR "atelidae"[Title/Abstract] OR "cottidae"[Title/Abstract] OR "leucopsis"[Title/Abstract] OR "acanthuridae"[Title/Abstract] OR "didelphimorphia"[Title/Abstract] OR "elver"[Title/Abstract] OR "elvers"[Title/Abstract] OR "lapponica"[Title/Abstract] OR "dermoptera"[Title/Abstract] OR "european hake"[Title/Abstract] OR "european hakes"[Title/Abstract] OR "gerbillinae"[Title/Abstract] OR "banteng"[Title/Abstract] OR "hartebeest"[Title/Abstract] OR "hartebeests"[Title/Abstract] OR "hogget"[Title/Abstract] OR "haematopus"[Title/Abstract] OR "anguis fragilis"[Title/Abstract] OR "grey heron"[Title/Abstract] OR "grey herons"[Title/Abstract] OR "blue whiting"[Title/Abstract] OR ("blue"[All Fields] AND "whitings"[Title/Abstract]) OR "furnariidae"[Title/Abstract] OR "macrovipera"[Title/Abstract] OR "esocidae"[Title/Abstract] OR "lapwing"[Title/Abstract] OR "lapwings"[Title/Abstract] OR "mylopharyngodon"[Title/Abstract] OR "wallabia"[Title/Abstract] OR "beloniformes"[Title/Abstract] OR "potoroo"[Title/Abstract] OR "potoroos"[Title/Abstract] OR "athene noctua"[Title/Abstract] OR "pleuronectidae"[Title/Abstract] OR "bushbabies"[Title/Abstract] OR "muscicapidae"[Title/Abstract] OR "alligatoridae"[Title/Abstract] OR "fuligula"[Title/Abstract] OR "bush baby"[Title/Abstract] OR "guineafowl"[Title/Abstract] OR "spoonbill"[Title/Abstract] OR "spoonbills"[Title/Abstract] OR "viverridae"[Title/Abstract] OR "catostomidae"[Title/Abstract] OR "zebrafishes"[Title/Abstract] OR "ibexes"[Title/Abstract] OR "vendace"[Title/Abstract] OR "estrildidae"[Title/Abstract] OR "monotremata"[Title/Abstract] OR "sepiella"[Title/Abstract] OR "ambystomatidae"[Title/Abstract] OR "shelduck"[Title/Abstract] OR "shelducks"[Title/Abstract] OR "treeshrew"[Title/Abstract] OR "treeshrews"[Title/Abstract] OR "hoplobatrachus"[Title/Abstract] OR "pochard"[Title/Abstract] OR "hoolock"[Title/Abstract] OR "hoolocks"[Title/Abstract] OR "lynxes"[Title/Abstract] OR "antilope"[Title/Abstract] OR "antilopes"[Title/Abstract] OR "blackbuck"[Title/Abstract] OR "blackbucks"[Title/Abstract] OR "cricetinae"[Title/Abstract] OR "paramisgurnus"[Title/Abstract] OR "skylark"[Title/Abstract] OR "skylarks"[Title/Abstract] OR "soleidae"[Title/Abstract] OR "allobates"[Title/Abstract] OR "northern wheatear"[Title/Abstract] OR "northern wheatears"[Title/Abstract] OR "pitheciidae"[Title/Abstract] OR "takin"[Title/Abstract] OR "theria"[Title/Abstract] OR "vanellus"[Title/Abstract] OR "galaxiidae"[Title/Abstract] OR "lorisidae"[Title/Abstract] OR "ostralegus"[Title/Abstract] OR "palaeognathae"[Title/Abstract] OR "stone loach"[Title/Abstract] OR "alauda"[Title/Abstract] OR "callitrichinae"[Title/Abstract] OR "caniformia"[Title/Abstract] OR "duttaphrynus"[Title/Abstract] OR "ictaluridae"[Title/Abstract] OR "osteoglossiformes"[Title/Abstract] OR "poultries"[Title/Abstract] OR "curema"[Title/Abstract] OR "ruddy turnstone"[Title/Abstract] OR "ruddy turnstones"[Title/Abstract] OR "sheatfish"[Title/Abstract] OR "sunfishes"[Title/Abstract] OR "centropomidae"[Title/Abstract] OR "hemachatus"[Title/Abstract] OR "platalea"[Title/Abstract] OR "thamnophilidae"[Title/Abstract] OR "song thrush"[Title/Abstract] OR "atherinopsidae"[Title/Abstract] OR "siluridae"[Title/Abstract] OR "tadorna"[Title/Abstract] OR "chroicocephalus"[Title/Abstract] OR "ermine"[Title/Abstract] OR "ermines"[Title/Abstract] OR "gavialis"[Title/Abstract] OR "ruff"[Title/Abstract] OR "tupaiidae"[Title/Abstract] OR "diprotodontia"[Title/Abstract] OR "hyaenidae"[Title/Abstract] OR "antilopinae"[Title/Abstract] OR "crocodylidae"[Title/Abstract] OR "herpestidae"[Title/Abstract] OR "hippopotamidae"[Title/Abstract] OR "northern shoveler"[Title/Abstract] OR "round gobies"[Title/Abstract] OR "cheirogaleidae"[Title/Abstract] OR "indriidae"[Title/Abstract] OR "fundulidae"[Title/Abstract] OR "pythonidae"[Title/Abstract] OR "rhynchocephalia"[Title/Abstract] OR "anodorhynchus"[Title/Abstract] OR "red backed shrike"[Title/Abstract] OR "red backed shrikes"[Title/Abstract] OR "triakidae"[Title/Abstract] OR "phalangeridae"[Title/Abstract] OR "aoudad"[Title/Abstract] OR "boreoeutheria"[Title/Abstract] OR "eurasian jay"[Title/Abstract] OR "eurasian jays"[Title/Abstract] OR "feliformia"[Title/Abstract] OR "haplorhini"[Title/Abstract] OR "osteoglossidae"[Title/Abstract] OR "paenungulata"[Title/Abstract] OR "struthioniformes"[Title/Abstract] OR "ferina"[Title/Abstract] OR "sanderling"[Title/Abstract] OR "sanderlings"[Title/Abstract] OR "spheniscidae"[Title/Abstract] OR "cuttlefishes"[Title/Abstract] OR "cygnet"[Title/Abstract] OR "dasycneme"[Title/Abstract] OR "gadwall"[Title/Abstract] OR "gadwalls"[Title/Abstract] OR "pelobates fuscus"[Title/Abstract] OR "wryneck"[Title/Abstract] OR "wrynecks"[Title/Abstract] OR "afrosoricida"[Title/Abstract] OR "culaea"[Title/Abstract] OR "dover sole"[Title/Abstract] OR ("dover"[All Fields] AND "soles"[Title/Abstract]) OR "paralichthyidae"[Title/Abstract] OR "passeridae"[Title/Abstract] OR "osteolaemus"[Title/Abstract] OR "song thrushes"[Title/Abstract] OR "bluethroat"[Title/Abstract] OR "bluethroats"[Title/Abstract] OR "hydrophiidae"[Title/Abstract] OR "megrim"[Title/Abstract] OR "mephitidae"[Title/Abstract] OR "strepsirhini"[Title/Abstract] OR "tomistoma"[Title/Abstract] OR "epidalea"[Title/Abstract] OR "osmeriformes"[Title/Abstract] OR "bush babies"[Title/Abstract] OR "tarsiiform"[Title/Abstract] OR "atelinae"[Title/Abstract] OR "bufotes"[Title/Abstract] OR "eurasian coot"[Title/Abstract] OR "eurasian coots"[Title/Abstract] OR "galagidae"[Title/Abstract] OR "geopelia"[Title/Abstract] OR "philomachus"[Title/Abstract] OR "tubulidentata"[Title/Abstract] OR "bombinatoridae"[Title/Abstract] OR "pelobatidae"[Title/Abstract] OR "tachysurus"[Title/Abstract] OR "ailuridae"[Title/Abstract] OR "woodlark"[Title/Abstract] OR "woodlarks"[Title/Abstract] OR "alcelaphinae"[Title/Abstract] OR "redshank"[Title/Abstract] OR "redshanks"[Title/Abstract] OR "salientia"[Title/Abstract] OR "sand smelt"[Title/Abstract] OR "sand smelts"[Title/Abstract] OR "woodmice"[Title/Abstract] OR "woodmouse"[Title/Abstract] OR "dasyproctidae"[Title/Abstract] OR "eurasian wigeon"[Title/Abstract] OR "eurasian wigeons"[Title/Abstract] OR "garganey"[Title/Abstract] OR "garganeys"[Title/Abstract] OR "lemon sole"[Title/Abstract] OR (("citrus"[MeSH Terms] OR "citrus"[All Fields] OR "lemon"[All Fields] OR "lemons"[All Fields]) AND "soles"[Title/Abstract]) OR "common dab"[Title/Abstract] OR (("common"[All Fields] OR "commonalities"[All Fields] OR "commonality"[All Fields] OR "commoner"[All Fields] OR "commoners"[All Fields] OR "commons"[All Fields]) AND "dabs"[Title/Abstract]) OR "graylag"[Title/Abstract] OR "graylags"[Title/Abstract] OR "leucorodia"[Title/Abstract] OR "osphronemidae"[Title/Abstract] OR "bewickii"[Title/Abstract] OR "common moorhen"[Title/Abstract] OR "common moorhens"[Title/Abstract] OR "decapodiformes"[Title/Abstract] OR "gobbler"[Title/Abstract] OR "gobblers"[Title/Abstract] OR "odontophoridae"[Title/Abstract] OR "paddlefishes"[Title/Abstract] OR "eutheria"[Title/Abstract] OR "salmonine"[Title/Abstract] OR "esociformes"[Title/Abstract] OR "eurasian woodcock"[Title/Abstract] OR "eurasian woodcocks"[Title/Abstract] OR "european smelt"[Title/Abstract] OR (("european people"[MeSH Terms] OR ("european"[All Fields] AND "people"[All Fields]) OR "european people"[All Fields] OR "european"[All Fields] OR "europeans"[All Fields]) AND "smelts"[Title/Abstract]) OR "goldfishes"[Title/Abstract] OR "tenches"[Title/Abstract] OR "tyranni"[Title/Abstract] OR "common chaffinch"[Title/Abstract] OR "common redstart"[Title/Abstract] OR "common redstarts"[Title/Abstract] OR "common roach"[Title/Abstract] OR (("common"[All Fields] OR "commonalities"[All Fields] OR "commonality"[All Fields] OR "commoner"[All Fields] OR "commoners"[All Fields] OR "commons"[All Fields]) AND "roachs"[Title/Abstract]) OR "great knot"[Title/Abstract] OR "great knots"[Title/Abstract] OR "potoroidae"[Title/Abstract] OR "alytidae"[Title/Abstract] OR "coregonine"[Title/Abstract] OR "dipteral"[Title/Abstract] OR "leveret"[Title/Abstract] OR "poeciliopsis gracilis"[Title/Abstract] OR "amphiumidae"[Title/Abstract] OR "batrachoidiformes"[Title/Abstract] OR "bighead goby"[Title/Abstract] OR "heteropneustidae"[Title/Abstract] OR "lullula"[Title/Abstract] OR "norway pout"[Title/Abstract] OR (("norway"[MeSH Terms] OR "norway"[All Fields] OR "norway s"[All Fields] OR "norways"[All Fields]) AND "pouts"[Title/Abstract]) OR "sipunculida"[Title/Abstract] OR "dogfishes"[Title/Abstract] OR "sebastidae"[Title/Abstract] OR "tarsiidae"[Title/Abstract] OR "alethinophidia"[Title/Abstract] OR "common nase"[Title/Abstract] OR (("common"[All Fields] OR "commonalities"[All Fields] OR "commonality"[All Fields] OR "commoner"[All Fields] OR "commoners"[All Fields] OR "commons"[All Fields]) AND "nases"[Title/Abstract]) OR "common sandpiper"[Title/Abstract] OR "common sandpipers"[Title/Abstract] OR "eurasian blackcap"[Title/Abstract] OR "eurasian blackcaps"[Title/Abstract] OR "pterocnemia"[Title/Abstract] OR "syngnathiformes"[Title/Abstract] OR "common chaffinches"[Title/Abstract] OR "eupleridae"[Title/Abstract] OR "octopodiformes"[Title/Abstract] OR "phascolarctidae"[Title/Abstract] OR "scophthalmidae"[Title/Abstract] OR "starry smooth hound"[Title/Abstract] OR ("starry"[All Fields] AND "smooth-hounds"[Title/Abstract]) OR "whitefishes"[Title/Abstract] OR "cuniculidae"[Title/Abstract] OR "european sprat"[Title/Abstract] OR (("european people"[MeSH Terms] OR ("european"[All Fields] AND "people"[All Fields]) OR "european people"[All Fields] OR "european"[All Fields] OR "europeans"[All Fields]) AND "sprats"[Title/Abstract]) OR "rosy bitterling"[Title/Abstract] OR ("rosy"[All Fields] AND "bitterlings"[Title/Abstract]) OR "common dace"[Title/Abstract] OR (("common"[All Fields] OR "commonalities"[All Fields] OR "commonality"[All Fields] OR "commoner"[All Fields] OR "commoners"[All Fields] OR "commons"[All Fields]) AND "daces"[Title/Abstract]) OR "lesser weever"[Title/Abstract] OR ("lesser"[All Fields] AND "weevers"[Title/Abstract]) OR "scaldfish"[Title/Abstract] OR "water rail"[Title/Abstract] OR "water rails"[Title/Abstract] OR "alouattinae"[Title/Abstract] OR "centrarchiformes"[Title/Abstract] OR "common whitethroat"[Title/Abstract] OR (("common"[All Fields] OR "commonalities"[All Fields] OR "commonality"[All Fields] OR "commoner"[All Fields] OR "commoners"[All Fields] OR "commons"[All Fields]) AND "whitethroats"[Title/Abstract]) OR "gavialidae"[Title/Abstract] OR "grey gurnard"[Title/Abstract] OR (("gray"[All Fields] OR "grey"[All Fields]) AND "gurnards"[Title/Abstract]) OR "lateolabracidae"[Title/Abstract] OR "rheiformes"[Title/Abstract] OR "tub gurnard"[Title/Abstract] OR ("tub"[All Fields] AND "gurnards"[Title/Abstract]) OR "common chiffchaff"[Title/Abstract] OR (("common"[All Fields] OR "commonalities"[All Fields] OR "commonality"[All Fields] OR "commoner"[All Fields] OR "commoners"[All Fields] OR "commons"[All Fields]) AND "chiffchaffs"[Title/Abstract]) OR "garfishes"[Title/Abstract] OR "lesser whitethroat"[Title/Abstract] OR ("lesser"[All Fields] AND "whitethroats"[Title/Abstract]) OR "myoxidae"[Title/Abstract] OR "seabasses"[Title/Abstract] OR "spariformes"[Title/Abstract] OR "umbridae"[Title/Abstract] OR "yellow boxfish"[Title/Abstract] OR "anabantiformes"[Title/Abstract] OR "aotidae"[Title/Abstract] OR "common bleak"[Title/Abstract] OR (("common"[All Fields] OR "commonalities"[All Fields] OR "commonality"[All Fields] OR "commoner"[All Fields] OR "commoners"[All Fields] OR "commons"[All Fields]) AND "bleaks"[Title/Abstract]) OR "common rudd"[Title/Abstract] OR (("common"[All Fields] OR "commonalities"[All Fields] OR "commonality"[All Fields] OR "commoner"[All Fields] OR "commoners"[All Fields] OR "commons"[All Fields]) AND "rudds"[Title/Abstract]) OR "greater pipefish"[Title/Abstract] OR "hapale"[Title/Abstract] OR "nandiniidae"[Title/Abstract] OR "stone loaches"[Title/Abstract] OR "whinchat"[Title/Abstract] OR "whinchats"[Title/Abstract] OR "acanthuriformes"[Title/Abstract] OR "brotula barbata"[Title/Abstract] OR "common ling"[Title/Abstract] OR (("common"[All Fields] OR "commonalities"[All Fields] OR "commonality"[All Fields] OR "commoner"[All Fields] OR "commoners"[All Fields] OR "commons"[All Fields]) AND "lings"[Title/Abstract]) OR (("common"[All Fields] OR "commonalities"[All Fields] OR "commonality"[All Fields] OR "commoner"[All Fields] OR "commoners"[All Fields] OR "commons"[All Fields]) AND "roaches"[Title/Abstract]) OR "cottonrat"[Title/Abstract] OR "cottonrats"[Title/Abstract] OR "douroucoulis"[Title/Abstract] OR "dromaiidae"[Title/Abstract] OR "fitches"[Title/Abstract] OR "fitchew"[Title/Abstract] OR "galaxiiformes"[Title/Abstract] OR "laprine"[Title/Abstract] OR "saimiriinae"[Title/Abstract] OR "solenette"[Title/Abstract] OR "tarsii"[Title/Abstract] OR "tompot blenny"[Title/Abstract] OR (("common"[All Fields] OR "commonalities"[All Fields] OR "commonality"[All Fields] OR "commoner"[All Fields] OR "commoners"[All Fields] OR "commons"[All Fields]) AND "dragonet"[Title/Abstract]) OR (("common"[All Fields] OR "commonalities"[All Fields] OR "commonality"[All Fields] OR "commoner"[All Fields] OR "commoners"[All Fields] OR "commons"[All Fields]) AND "dragonets"[Title/Abstract]) OR ("longspined"[All Fields] AND "bullhead"[Title/Abstract]) OR ("longspined"[All Fields] AND "bullheads"[Title/Abstract]) OR "monotremate"[Title/Abstract] OR "pempheriformes"[Title/Abstract] OR "perdicinae"[Title/Abstract] OR "presbytini"[Title/Abstract] OR "smegmamorpha"[Title/Abstract] OR (("bighead"[All Fields] OR "bigheaded"[All Fields]) AND "gobies"[Title/Abstract]) OR ("carangaria"[All Fields] AND "incertae sedis"[Title/Abstract]) OR ("rockling"[Title/Abstract]) OR ("greater"[All Fields] AND "pipefishes"[Title/Abstract]) OR (("norwegian"[All Fields] OR "norwegians"[All Fields]) AND "topknot"[Title/Abstract]) OR (("indian ocean"[MeSH Terms] OR ("indian"[All Fields] AND "ocean"[All Fields]) OR "indian ocean"[All Fields] OR ("red"[All Fields] AND "sea"[All Fields]) OR "red sea"[All Fields]) AND "sailfin tang"[Title/Abstract]) OR "sheatfishes"[Title/Abstract] OR ("tompot"[All Fields] AND "blennies"[Title/Abstract]) OR ("twait"[All Fields] AND "shad"[Title/Abstract]) OR (("yellow"[All Fields] OR "yellowed"[All Fields] OR "yellower"[All Fields] OR "yellowing"[All Fields] OR "yellowness"[All Fields] OR "yellows"[All Fields]) AND "boxfishes"[Title/Abstract])) NOT "humans"[MeSH Terms]))

## Web of Science

TI = ((alzheimer* OR amyloid OR tau) AND (model* OR simulat* OR 'in silico' OR 'quantitative systems pharmacology')) AND AB = ((math* OR comput* OR "in silico" OR "quantitative systems pharmacology" OR QSP OR pharmacokinetic OR pharmacodynamic) AND (antibod* OR therap* OR drug OR intervention* OR agent*) ) NOT TS = ((rat OR rats OR animal OR animals OR mice OR mouse OR rabbit OR rabbits OR murine OR pig OR pigs OR dog OR dogs OR bovine OR fish OR vertebrate OR vertebrates OR cat OR cats OR rodent OR rodents OR mammal OR mammals OR chicken OR chickens OR monkey OR monkeys OR sheep OR canine OR canines OR porcine OR cattle OR bird OR birds OR hamster OR hamsters OR primate OR primates OR cow OR cows OR chick OR horse OR horses OR avian OR avians OR calf OR swine OR swines OR xenopus OR turkeys OR bear OR bears OR frog OR frogs OR zebrafish OR goat OR goats OR equine OR calves OR poultry OR macaque OR macaques OR mole OR moles OR ovine OR lamb OR lambs OR fishes OR diptera OR amphibian OR amphibians OR snake OR snakes OR ruminant OR ruminants OR hen OR hens OR piglet OR piglets OR feline OR felines OR simian OR simians OR laevis OR trout OR trouts OR teleost OR teleosts salmon OR salmons OR seal OR seals OR bull OR bulls OR ewe OR ewes OR hedgehog OR hedgehogs OR macaca OR macacas OR proteus OR pigeon OR pigeons OR bat OR bats OR duck OR ducks OR chimpanzee OR chimpanzees OR baboon OR baboons OR deer OR rana OR ranas OR carp OR carps OR heifer OR swallow OR swallows OR lizard OR lizards OR canis OR sow OR sows OR cynomolgus OR quail OR quails OR reptile OR reptiles OR turtle OR turtles OR buffalo OR gerbil OR gerbils OR boar OR boars OR squirrel OR squirrels OR oncorhynchus OR mus OR toad OR toads OR fowl OR fowls OR rerio OR danio OR ara OR aras OR musculus OR tadpole OR tadpoles OR mulatta OR salmo OR ram OR eagle OR eagles OR ferret OR ferrets OR goldfish OR catfish OR whale OR whales OR fox OR foxes OR ape OR apes OR elephant OR elephants OR bos OR marmoset OR marmosets OR cod OR cods OR shark OR sharks OR wolf OR eel OR eels OR auratus OR rattus OR zebra OR zebras OR tilapia OR tilapias OR gilt OR camel OR camels OR squid OR gallus OR marsupial OR marsupials OR vole OR voles OR fascicularis OR ovis OR salmonid OR salmonids OR tiger OR tigers OR dolphin OR dolphins OR robin OR robins OR carpio OR opossum OR opossums OR cyprinus OR salamander OR salamanders OR felis OR mink OR minks OR swan OR swans OR norvegicus OR bufo OR torpedo OR bass OR lamprey OR lampreys OR sus OR python OR pythons OR tetrapod OR tetrapods OR shrew OR shrews OR lion OR lions OR hog OR hogs OR songbird OR songbirds OR oreochromis OR starling OR starlings OR caprine OR carassius OR owl OR owls OR newt OR newts OR papio OR scrofa OR hare OR hares OR gorilla OR gorillas OR flounder OR flounders OR goose OR herring OR herrings OR therian OR buffaloes OR canary OR sparrow OR sparrows O Rmicrotus OR octopus OR troglodytes OR tuna OR amphibia OR chinchilla OR chinchillas OR ide OR oryzias OR cervus OR kangaroo OR kangaroos OR armadillo OR armadillos OR callithrix OR "pan troglodytes" OR saimiri OR cichlid OR cichlids OR donkey OR donkeys OR bream OR char OR chars OR finch OR raccoon OR raccoons OR bothrops OR anguilla OR perch OR cricetus OR seabird OR seabirds OR buck OR bucks OR naja OR coturnix OR salmonids OR geese OR minnow OR minnows OR raptor OR raptors OR merione OR meriones OR rodentia OR elaphus OR amniote OR amniotes OR elasmobranch OR emu OR emus OR peromyscus OR hominid OR hominids OR bubalus OR crotalus OR gull OR gulls OR anas OR anura OR lemur OR lemurs OR crow OR crows OR camelus OR gibbon OR gibbons OR waterfowl OR parrot OR parrots OR eels OR cob OR stickleback OR sticklebacks OR columba OR mesocricetus OR ambystoma OR raven OR ravens OR gadus OR penguin OR penguins OR orangutan OR orangutans OR sturgeon OR sturgeons OR cuniculus OR aves OR virginianus OR cephalopod OR cephalopods OR cebus OR sparus OR tortoise OR tortoises OR guttata OR morhua OR unguiculatus OR dogfish OR vulpes OR mallard OR mallards OR apodemus OR alligator OR alligators OR oryctolagus OR llama OR llamas OR reindeer OR mustela OR duckling OR ducklings OR wolves OR sander OR amazona OR zebu OR badger OR badgers OR dove OR doves OR ictalurus OR capra OR capras OR equus OR camelid OR camelids OR poecilia OR mule OR mules OR perciformes OR salvelinus OR labrax OR cyprinidae OR ariidae OR crocodile OR crocodiles OR fundulus OR dicentrarchus OR clarias OR cercopithecus OR chiroptera OR alpaca OR alpacas OR pike OR pikes OR paralichthys OR puma OR pumas OR didelphis OR pisces OR macropus OR triturus OR bison OR bisons OR epinephelus OR gasterosteus OR panthera OR acipenser OR mackerel OR mackerels OR tamarin OR tamarins OR ostrich OR anolis OR vervet OR vervets OR wallaby OR glareolus OR beaver OR beavers OR dromedary OR catus OR killifish OR pimephales OR promelas OR aotus OR phoca OR panda OR pandas OR porpoise OR porpoises OR myotis OR yak OR yaks OR agkistrodon OR vipera OR otter OR otters OR turbot OR turbots OR squamate OR carnivora OR mullet OR mullets OR hawk OR hawks OR taeniopygia OR seahorse OR seahorses OR "poecilia reticulata" OR falcon OR falcons OR prosimian OR prosimians OR parus OR perca OR fingerling OR fingerlings OR antelope OR antelopes OR tupaia OR passeriformes OR sepia OR saguinus OR coyote OR coyotes OR pongo OR meleagris OR reptilia OR lepus OR psittacine OR hagfish OR warbler OR warblers OR "russell s viper" OR "russell s vipers" OR smolt OR smolts OR budgerigar OR sardine OR sardines OR cavia OR cavias OR hyla OR pleurodeles OR siluriformes OR "great tit" OR "great tits" OR guppy OR bonobo OR bonobos OR rutilus OR trichosurus OR muridae OR phodopus OR channa OR squalus OR lynx OR sturnus OR petromyzon OR vitulina OR monodelphis OR cuttlefish OR adder OR adders OR lepomis OR canaria OR gambusia OR guppies OR xiphophorus OR flatfish OR koala OR koalas OR labeo OR stingray OR stingrays OR chelonia OR lampetra OR spermophilus OR crocodilian OR "passer domesticus" OR sciurus OR artiodactyla OR ranidae OR corvus OR necturus OR platypus OR canaries OR bovid OR lagopus OR trimeresurus OR gariepinus OR marten OR martens OR drosophilidae OR mugil OR sunfish OR porcellus OR cypriniformes OR alouatta OR scophthalmus OR anser OR electrophorus OR putorius OR iguana OR iguanas OR lama OR lamas OR takifugu OR circus OR eptesicus OR flycatcher OR galago OR galagos OR trachemys OR lungfish OR characiformes OR shorebird OR shorebirds OR giraffe OR giraffes OR micropterus OR scyliorhinus OR cichlidae OR loligo OR porcupine OR porcupines OR chub OR chubs OR solea OR pleuronectes OR hylidae OR viperidae OR echis OR sorex OR anchovy OR lagomorph OR ostriches OR vulture OR vultures OR whitefish OR araneus OR jird OR jirds OR tern OR esox OR drake OR drakes OR elapidae OR gallopavo OR chordata OR myodes OR caretta OR serinus OR grouse OR misgurnus OR meles OR blackbird OR blackbirds OR coregonus OR bobwhite OR bobwhites OR heteropneustes OR mammoth OR mammoths OR turdus OR rhinella OR ateles OR characidae OR clupea OR bungarus OR brill OR "struthiocamelus" OR sloth OR sloths OR pteropus OR sculpin OR anthropoids OR pollock OR pollocks OR morone OR "pan paniscus" OR litoria OR chipmunk OR chipmunks OR balaenoptera OR marmota OR melopsittacus OR hyrax OR lemming OR lemmings OR halibut OR hylobates OR lates OR caiman OR caimans OR sigmodon OR stenella OR barbel OR barbels OR sterna OR parakeet OR parakeets OR phocoena OR leptodactylus OR canidae OR buteo OR harengus OR gopher OR gophers OR marmot OR marmots OR gosling OR goslings OR platichthys OR gar OR gars OR sebastes OR marsupialia OR notophthalmus OR gazelle OR gazelles OR insectivora OR paridae OR felidae OR russula OR galliformes OR bombina OR colobus OR echidna OR echidnas OR seabass OR syncerus OR plaice OR "blue tit" OR "blue tits" OR pagrus OR catfishes OR cetacea OR barbus OR cygnus OR ficedula OR chamois OR colubridae OR perches OR coelacanth OR fitch OR urodela OR cynops OR martes OR halichoerus OR aix OR salmonidae OR leuciscus OR magpie OR magpies OR silurus OR whiting OR whitings OR anseriformes OR colinus OR rhea OR chlorocebus OR octodon OR acinonyx OR mouflon OR mouflons OR ibex OR tetraodon OR bufonidae OR equidae OR jackal OR cephalopoda OR dendroaspis OR glama OR muskrat OR muskrats OR sable OR sables OR wildebeest OR streptopelia OR albifrons OR vespertilionidae OR woodpecker OR woodpeckers OR muntjac OR muntjacs OR archosaur OR branta OR cricetulus OR megalobrama OR poeciliidae OR desmodus OR snakehead OR snakeheads OR tench OR teal OR teals OR bandicoot OR bandicoots OR apteronotus OR phyllostomidae OR crocidura OR buzzard OR buzzards OR larimichthys OR cercocebus OR pipistrellus OR erithacus OR impala OR impalas OR rousettus OR haddock OR haddocks OR tinca OR ratite OR calidris OR cynoglossus OR hypophthalmichthys OR bullock OR bullocks OR dromedaries OR alectoris OR filly OR salamandra OR cingulata OR bitis OR grus OR ammodytes OR macaw OR macaws OR hypoleuca OR sapajus OR cyprinodontiformes OR hippopotamus OR pelophylax OR capybara OR capybaras OR weasel OR weasels OR cairina OR cynomys OR lutra OR cockatoo OR cockatoos OR lachesis OR lagomorpha OR rupicapra OR daboia OR "orang utan" OR "orang utans" OR platyrrhini OR charadriiformes OR micrurus OR psittaciformes OR spalax OR loris OR mustelidae OR sylvilagus OR vitticeps OR cockatiel OR mustelus OR cottus OR erythrocebus OR dipodomys OR platessa OR callicebus OR loricariidae OR catostomus OR cuneata OR cyanistes OR cyprinodon OR sigmodontinae OR elasmobranchii OR trichechus OR sauropsid OR xenarthra OR dormouse OR perissodactyla OR nautilus OR cirrhinus OR gulo OR gulos OR tragelaphus OR merula OR numida OR sciaenidae OR cerastes OR sciuridae OR gibbosus OR octopuses OR eland OR elands OR phyllomedusa OR pogona OR walrus OR agamidae OR leptodactylidae OR ridibundus OR leontopithecus OR anteater OR anteaters OR pelodiscus OR cebidae OR columbianus OR "pelteobagrus fulvidraco" OR hominoidea OR mandrillus OR "zonotrichia leucophrys" OR agama OR gobiocypris OR "bearded dragon" OR "bearded dragons" OR sarotherodon OR talpa OR discoglossus OR hagfishes OR sphenodon OR gudgeon OR amphiuma OR aythya OR tenrec OR tenrec OR hominidae OR risoria OR salamandridae OR camelidae OR columbiformes OR latimeria OR plover OR plovers OR afrotheria OR "falcosparverius" OR polecat OR polecats OR crotalinae OR salvadora OR tarsier OR lucioperca OR anchovies OR lungfishes OR terrapin OR "dromaius novaehollandiae" OR lateolabrax OR eigenmannia OR pelamis OR theropithecus OR murinae OR gander OR gymnotus OR pseudacris OR gymnophiona OR gymnotiformes OR laticauda OR falconiformes OR dugong OR dugongs OR pintail OR pintails OR rook OR rooks OR lasiurus OR catshark OR catsharks OR micropogonias OR "redjunglefowl" OR paddlefish OR eutheria OR ophiophagus OR hollandicus OR nymphicus OR pimelodidae OR aepyceros OR cobitidae OR strigiformes OR cobitis OR dormice OR alytes OR calloselasma OR guanaco OR guanacos OR phasianidae OR "round goby" OR trichogaster OR catarrhini OR eelpout OR eelpouts OR galaxias OR gaur OR pungitius OR suslik OR susliks OR flatfishes OR percidae OR caprinae OR todarodes OR osmerus OR ameiurus OR anthropoidea OR "castor canadensis" OR pouting OR poutings OR tetraodontiformes OR arvicolinae OR siamang OR siamangs OR "castor fiber" OR nomascus OR "redknot" OR "red knots" OR syngnathidae OR iguanidae OR eretmochelys OR ursidae OR callimico OR columbidae OR microhylidae OR anaxyrus OR menidia OR pipistrelle OR greylag OR pipidae OR scandentia OR bowfin OR bowfins OR dendrobatidae OR zenaida OR bushbaby OR harrier OR harriers OR macropodidae OR pygerythrus OR clupeidae OR odorrana OR corvidae OR jerboa OR jerboas OR canutus OR hylobatidae OR clupeiformes OR "great cormorant" OR "greatcormorants" OR scorpaeniformes OR chondrostean OR garfish OR proboscidea OR psetta OR diapsid OR serotinus OR tetrao OR walruses OR carcharhiniformes OR leucoraja OR pumpkinseed OR dosidicus OR acipenseriformes OR daubentonii OR emberizidae OR gadiformes OR hyraxes OR stizostedion OR wolverine OR wolverines OR lissotriton OR acanthurus OR centrarchidae OR gloydius OR laurasiatheria OR limosa OR psittacula OR leporidae OR proteidae OR zander OR zanders OR arapaima OR bagridae OR cyprinodontidae OR mithun OR pandion OR jackdaw OR jackdaws OR procyonidae OR carus OR jaculus OR salmoniformes OR "common sole" OR "common soles" OR protobothrops OR calamita OR brachyteles OR trionyx OR turdidae OR boidae OR luscinia OR pugnax OR euarchontoglires OR saithe OR saithes OR symphalangus OR aardvark OR aardvarks OR oystercatcher OR oystercatchers OR arius OR corydoras OR poacher OR poachers OR aurochs OR cebuella OR crecca OR lemuridae OR sirenia OR lemmus OR perdix OR glires OR lepidosaur OR muskox OR deinagkistrodon OR Pholidota OR holocephali OR cercopithecinae OR clariidae OR agapornis OR doryteuthis OR tyrannidae OR dicroglossidae OR godwit OR godwits OR monedula OR pongidae OR atheriniformes OR colobinae OR lophocebus OR atelidae OR cottidae OR leucopsis OR acanthuridae OR didelphimorphia OR elver OR elvers OR lapponica OR dermoptera OR "european hake" OR "european hakes" OR gerbillinae OR banteng OR hartebeest OR hartebeests OR hogget OR haematopus OR "anguis fragilis" OR "grey heron" OR "grey herons" OR "blue whiting" OR "blue whitings" OR furnariidae OR macrovipera OR esocidae OR lapwing OR lapwings OR mylopharyngodon OR wallabia OR beloniformes OR potoroo OR potoroos OR "athene noctua" OR pleuronectidae OR bushbabies OR muscicapidae OR alligatoridae OR fuligula OR "bush baby" OR guineafowl OR spoonbill OR spoonbills OR viverridae OR catostomidae OR zebrafishes OR ibexes OR vendace OR estrildidae OR monotremata OR sepiella OR ambystomatidae OR shelduck OR shelducks OR treeshrew OR treeshrews OR hoplobatrachus OR pochard OR hoolock OR hoolocks OR lynxes OR antilope OR antilopes OR blackbuck OR blackbucks OR cricetinae OR paramisgurnus OR skylark OR skylarks OR soleidae OR allobates OR "northern wheatear" OR "northern wheatears" OR pitheciidae OR takin OR theria OR vanellus OR galaxiidae OR lorisidae OR ostralegus OR palaeognathae OR "stone loach" OR alauda OR callitrichinae OR caniformia OR duttaphrynus OR ictaluridae OR osteoglossiformes OR poultries OR curema OR "ruddy turnstone" OR "ruddy turnstones" OR sheatfish OR sunfishes OR centropomidae OR hemachatus OR platalea OR thamnophilidae OR "songthrush" OR atherinopsidae OR siluridae OR tadorna OR chroicocephalus OR ermine OR ermines OR gavialis OR ruffe OR tupaiidae OR diprotodontia OR hyaenidae OR antilopinae OR crocodylidae OR herpestidae OR hippopotamidae OR "northern shoveler" OR "round gobies" OR cheirogaleidae OR indriidae OR fundulidae OR pythonidae OR rhynchocephalia OR anodorhynchus OR "red-backed shrike" OR "red-backed shrikes" OR triakidae OR phalangeridae OR aoudad OR boreoeutheria OR "eurasian jay" OR "eurasian jays" OR feliformia OR haplorhini OR osteoglossidae OR paenungulata OR struthioniformes OR ferina OR sanderling OR sanderlings OR spheniscidae OR cuttlefishes OR cygnet OR dasycneme OR gadwall OR gadwalls OR "pelobates fuscus" OR wryneck OR wrynecks OR afrosoricida OR culaea OR "dover sole" OR "doversoles" OR paralichthyidae OR passeridae OR osteolaemus OR "song thrushes" OR bluethroat OR bluethroats OR hydrophiidae OR megrim OR mephitidae OR strepsirhini OR tomistoma OR epidalea OR osmeriformes OR "bush babies" OR tarsiiform OR atelinae OR bufotes OR "eurasian coot" OR "eurasian coots" OR galagidae OR geopelia OR philomachus OR tubulidentata OR bombinatoridae OR pelobatidae OR tachysurus OR ailuridae OR woodlark OR woodlarks OR alcelaphinae OR redshank OR redshanks OR salientia OR "sand smelt" OR "sand smelts" OR woodmice OR woodmouse OR dasyproctidae OR "eurasian wigeon" OR "eurasian wigeons" OR garganey OR garganeys OR "lemon sole" OR "lemon soles" OR "commondab" OR "common dabs" OR graylag OR graylags OR leucorodia OR osphronemidae OR bewickii OR "common moorhen" OR "common moorhens" OR decapodiformes OR gobbler OR gobblers OR odontophoridae OR paddlefishes OR salmonine OR esociformes OR "eurasian woodcock" OR "eurasian woodcocks" OR "european smelt" OR "european smelts" OR goldfishes OR tenches OR tyranni OR "common chaffinch" OR "common chaffinchs" OR "common redstart" OR "common redstarts" OR "common roach" OR "common roachs" OR "great knot" OR "great knots" OR potoroidae OR alytidae OR coregonine OR dipteral OR leveret OR "poeciliopsis gracilis" OR amphiumidae OR batrachoidiformes OR "bighead goby" OR heteropneustidae OR lullula OR "norway pout" OR "norway pouts" OR sipunculida OR dogfishes OR sebastidae OR tarsiidae OR alethinophidia OR "common nase" OR "common nases" OR "common sandpiper" OR "common sandpipers" OR "eurasian blackcap" OR "eurasian blackcaps" OR pterocnemia OR syngnathiformes OR "common chaffinches" OR eupleridae OR octopodiformes OR phascolarctidae OR scophthalmidae OR "starry smooth-hound" OR "starry smooth-hounds" OR whitefishes OR cuniculidae OR "european sprat" OR "european sprats" OR "rosy bitterling" OR "rosy bitterlings" OR "common dace" OR "common daces" OR "lesser weever" OR "lesser weevers" OR scaldfish OR "water rail" OR "water rails" OR alouattinae OR centrarchiformes OR "common whitethroat" OR "common whitethroats" OR gavialidae OR "greygurnard" OR "grey gurnards" OR lateolabracidae OR rheiformes OR "tub gurnard" OR "tub gurnards" OR "commonchiffchaff" OR "common chiffchaffs" OR garfishes OR "lesser whitethroat" OR "lesser whitethroats" OR myoxidae OR seabasses OR spariformes OR umbridae OR "yellow boxfish" OR anabantiformes OR aotidae OR "common bleak" OR "common bleaks" OR "common rudd" OR "common rudds" OR "greater pipefish" OR hapale OR nandiniidae OR "stoneloaches" OR whinchat OR whinchats OR acanthuriformes OR "brotula barbata" OR "common ling" OR "common lings" OR "common roaches" OR cottonrat OR cottonrats OR douroucoulis OR dromaiidae OR fitches OR fitchew OR galaxiiformes OR laprine OR saimiriinae OR solenette OR tarsii OR "tompot blenny" OR "common dragonet" OR "common dragonets" OR "longspined bullhead" OR "longspined bullheads" OR monotremate OR monotremates OR pempheriformes OR perdicinae OR presbytini OR smegmamorpha OR "bighead gobies" OR "carangaria incertae sedis" OR coiidae OR "fivebeard rockling" OR foulmart OR foumart OR grasskeet OR "greater pipefishes" OR ibices OR millionfish OR muguliformes OR "norwegiantopknot" OR peewit OR "red sea sailfin tang" OR rupicapras OR sheatfishes OR "tompot blennies" OR "twait shad" OR "yellow boxfishes") NOT human)

# *Complete screening summary statistics*

Annotations for all literature included in the review are provided in Supplementary Table S1, including the decision, the screening stage at which the decision was made, and the reason for this decision for all papers excluded during the title and abstract screening stages.

Further detail on the inclusion criteria missed by each paper at the full-text screening stage, alongside descriptive notes to explain the decisions, are provided in Supplementary Table S2.

The information below summarises the number of publications excluded at each stage and their reasons. The reason codes applied through Rayyan, and listed in Supplementary Tables S1 and S2, are stated in parenthesis below.

Title screening: 540 → 283

257 publications excluded:

- 179 for molecular modelling approaches (‘wrong study design”)
- 46 for being ‘animal’ experimental models
- 8 for being machine learning
- 5 for being reviews (“background article”)
- 4 for being ‘in vitro’ experimental models
- 4 for being conference proceedings (“wrong publication type”)
- 4 for modelling other amyloids e.g., iAPP (“wrong population”)
- 4 for being bioinformatics studies
- 1 for modelling cancer
- 1 model of electrode position for transcranial direct current stimulation
- 1 publication describing a virtual patient journal

Abstract screening: 283 → 91

192 publications excluded:

- 149 for molecular modelling approaches (‘wrong study design”)
- 25 for being reviews (“background article”)
- 5 for being bioinformatics studies
- 4 for being ‘in vitro’ experimental models
- 2 for modelling other amyloids e.g., iAPP (“wrong population”)
- 2 for machine learning
- 1 for literature mining
- 1 for the paper being inaccessible
- 1 for modelling disease progression without treatment
- 1 for modelling implant design
- 1 for simulating cholinergic treatments only

Full-text screening: 91 → 7

84 publications excluded:

- 3 reviews of background articles (“background article”)
- 3 epidemiological models
- 3 in vitro models
- 3 for molecular modelling approaches (‘wrong study design”)
- 1 neural network
- 1 for being bioinformatic study
- 70 other (see Supplementary Table 2)

Details on the systems models with clinically relevant outputs, but without treatments represented in a way that meets the inclusion criteria, can be found in Supplementary Table S3. We hope this resource can act as a reference for the development of future QSP models, allowing existing mathematical models of AD to be expanded for application in new pharmacological settings.

# *Summaries of the seven included anti-amyloid QSP models*

Model 1: A combined physiologically-based pharmacokinetic and quantitative systems pharmacology model for modeling amyloid aggregation in Alzheimer's disease (Geerts et al. 2023)^1^

- A QSP model is developed to link imaging and fluid biomarkers to the biological pathways central to the amyloid hypothesis in order to better understand the effects of six anti-amyloid monoclonal antibodies. Specifically, amyloid clearance and ARIA-E incidence are modelled for the various drugs, and the effect of the APOEe4 allele on both ARIA-E and amyloid clearance is included. Amyloid PET SUVr lowering is used as a surrogate for clinical benefit, and is modelled through clearance of all Aβ aggregates (oligomers, protofibrils, and plaque).

Model 2: Mechanistic Modeling of Soluble Aβ Dynamics and Target Engagement in the Brain by Anti-Aβ mAbs in Alzheimer's Disease (Ferl et al. 2020)^4^

- A QSP model developed to facilitate comparison between anti-amyloid monoclonal antibodies with different target engagement profiles. Two existing antibodies were compared at various doses, and the effect of a hypothetical increase in brain penetration was simulated for both. The main model output is oligomer clearance, on the basis of the hypothesis that Aβ oligomers are the primary neurotoxic species.

Model 3: Passive Immunotherapies Targeting Amyloid-β in Alzheimer's Disease: A Quantitative Systems Pharmacology Perspective (Markovic et al. 2024)^5^

- A QSP model to model the effects of monomer-binding monoclonal antibodies on Aβ aggregation and plaque clearance and investigate whether alterations to the treatment protocol could make such drugs effective. Hypothetical monoclonal antibodies with broad Aβ affinity were also modelled. Those with and without effector function compared with respect to plaque clearance.

Model 4: Quantitative systems pharmacology model for Alzheimer's disease to predict the effect of aducanumab on brain amyloid (Lin et al. 2022)^6^

- A QSP model to simulate the effect of aducanumab on the Aβ pathways in AD. Having developed the model based on existing clinical trial data for aducanumab, it was used to simulate alternative dosing regimens and treatment protocols, including over longer timeframes than the trial period. Amyloid PET SUVr lowering is used as a surrogate for clinical benefit, and is modelled through clearance of Aβ plaque.

Model 5: Quantitative systems pharmacology model of the amyloid pathway in Alzheimer's disease: Insights into the therapeutic mechanisms of clinical candidates (Ramakrishnan et al. 2023)^7^

- Structurally similar to Lin et al. 2022, this model differs from previously developed QSP models by separately modelling Aβ40 and Aβ42 at the monomer level, capturing their different aggregation and clearance rates and how they are targeted by different monoclonal antibodies. The model is also used to investigate differences in treatment response between APOEe4 carriers and non-carriers. Amyloid PET SUVr lowering is used as a surrogate for clinical benefit, and is modelled through clearance of Aβ fibrils and plaque.

Model 6: Quantitative systems pharmacology-based exploration of relevant anti-amyloid therapy challenges in clinical practice (Geerts et al. 2024)

- Using the QSP model in 1, here an additional monoclonal antibody, donanemab, is simulated. In addition, possible maintenance treatment regimens are investigated, and the model is adapted to the Down Syndrome population to compare treatment effects and ARIA-E incidence with the sporadic AD population.

Model 7: Systematic in silico analysis of clinically tested drugs for reducing amyloid-beta plaque accumulation in Alzheimer's disease (Madrasi et al. 2021)^9^

- A QSP model for a wide range of anti-amyloid therapeutics including beta-secretase and gamma-secretase inhibitors, and monoclonal antibodies. As such, it can compare the potential of targeting the peripheral sink hypothesis vs driving plaque clearance through ADCP. Amyloid PET SUVr lowering is used as a surrogate for clinical benefit, and is modelled through clearance of all Aβ plaque.

# *Conversion of fractional rates*

Model 2: Amyloid-beta transport parameters are represented as clearance rates with units /d. Therefore, the following conversions were applied:

Aβ brain clearance = 2.9 /d = 0.000336 /s

Aβ plasma clearance = 82 /d = 0.00949 /s

Aβ brain → plasma = 0.25 * (Aβ brain clearance) = 0.0000839 /s

Aβ brain → CSF = 0.25 * (Aβ brain clearance) = 0.0000839 /s

Model 4: All parameters listed are given in units /s, therefore no scaling was required.

Model 5: Amyloid-beta transport parameters are represented as clearance rates with units /h. Therefore, the following conversions were applied:

Aβ brain clearance = 0.0356 /h = 0. 0000989/s

Aβ plasma clearance = 0.231 /h = 0.000642 /s

Aβ brain → plasma = 0.246 * (Aβ brain clearance) = 0.0000243 /s

Aβ brain → CSF = 0.353 * (Aβ brain clearance) = 0.0000349 /s

Model 7: All parameters listed are given in units /s, therefore no scaling was required.

# *List of Supplementary Tables*

- - Table S1: Log of all literature screened.
  - Table S2: Inclusion/exclusion details for full-text screening.
  - Table S3: Summaries of models excluded during full-text screening with clinically relevant outputs.
  - Table S4: Data extracted from the seven included studies.
  - Table S5: Model quality scores.
  - Table S6: Comparison of antibody affinities to different amyloid species used in modelling.

# *References*

1. Geerts H, Walker M, Rose R, et al. A combined physiologically-based pharmacokinetic and quantitative systems pharmacology model for modeling amyloid aggregation in Alzheimer’s disease. *CPT Pharmacomet Syst Pharmacol*. 2023;12(4):444-461. doi:10.1002/psp4.12912

2. Geerts H, Spiros A, Roberts P. Impact of amyloid-beta changes on cognitive outcomes in Alzheimer’s disease: analysis of clinical trials using a quantitative systems pharmacology model. *ALZHEIMERS Res Ther*. 2018;10. doi:10.1186/s13195-018-0343-5

3. Geerts H, Spiros A. Learning from amyloid trials in Alzheimer’s disease. A virtual patient analysis using a quantitative systems pharmacology approach. *Alzheimers Dement*. 2020;16(6):862-872. doi:10.1002/alz.12082

4. Ferl GZ, Fuji RN, Atwal JK, Sun T, Ramanujan S, Quartino AL. Mechanistic Modeling of Soluble Aβ Dynamics and Target Engagement in the Brain by Anti-Aβ mAbs in Alzheimer’s Disease. *Curr Alzheimer Res*. 2020;17(4):393-406. doi:10.2174/1567205017666200302122307

5. Marković M, Milošević J, Wang W, Cao Y. Passive Immunotherapies Targeting Amyloid-β in Alzheimer’s Disease: A Quantitative Systems Pharmacology Perspective. *Mol Pharmacol*. 2024;105(1):1-13. doi:10.1124/molpharm.123.000726

6. Lin L, Hua F, Salinas C, et al. Quantitative systems pharmacology model for Alzheimer’s disease to predict the effect of aducanumab on brain amyloid. *CPT Pharmacomet Syst Pharmacol*. 2022;11(3):362-372. doi:10.1002/psp4.12759

7. Ramakrishnan V, Friedrich C, Witt C, et al. Quantitative systems pharmacology model of the amyloid pathway in Alzheimer’s disease: Insights into the therapeutic mechanisms of clinical candidates. *CPT Pharmacomet Syst Pharmacol*. 2023;12(1):62-73. doi:10.1002/psp4.12876

8. Geerts H, Bergeler S, Walker M, Rose RH, van der Graaf PH. Quantitative systems pharmacology-based exploration of relevant anti-amyloid therapy challenges in clinical practice. *Alzheimers Dement Transl Res Clin Interv*. 2024;10(2):e12474. doi:10.1002/trc2.12474

9. Madrasi K, Das R, Mohmmadabdul H, et al. Systematic in silico analysis of clinically tested drugs for reducing amyloid-beta plaque accumulation in Alzheimer’s disease. *Alzheimers Dement*. 2021;17(9):1487-1498. doi:10.1002/alz.12312
